# Measurement of the inclusive and fiducial $t\bar{t}$ production cross-sections in the lepton+jets channel in *pp* collisions at $\sqrt{s} = 8~\hbox {TeV}$ with the ATLAS detector

**DOI:** 10.1140/epjc/s10052-018-5904-z

**Published:** 2018-06-12

**Authors:** M. Aaboud, G. Aad, B. Abbott, O. Abdinov, B. Abeloos, S. H. Abidi, O. S. AbouZeid, N. L. Abraham, H. Abramowicz, H. Abreu, R. Abreu, Y. Abulaiti, B. S. Acharya, S. Adachi, L. Adamczyk, J. Adelman, M. Adersberger, T. Adye, A. A. Affolder, Y. Afik, T. Agatonovic-Jovin, C. Agheorghiesei, J. A. Aguilar-Saavedra, S. P. Ahlen, F. Ahmadov, G. Aielli, S. Akatsuka, H. Akerstedt, T. P. A. Åkesson, E. Akilli, A. V. Akimov, G. L. Alberghi, J. Albert, P. Albicocco, M. J. Alconada Verzini, S. C. Alderweireldt, M. Aleksa, I. N. Aleksandrov, C. Alexa, G. Alexander, T. Alexopoulos, M. Alhroob, B. Ali, M. Aliev, G. Alimonti, J. Alison, S. P. Alkire, B. M. M. Allbrooke, B. W. Allen, P. P. Allport, A. Aloisio, A. Alonso, F. Alonso, C. Alpigiani, A. A. Alshehri, M. I. Alstaty, B. Alvarez Gonzalez, D. Álvarez Piqueras, M. G. Alviggi, B. T. Amadio, Y. Amaral Coutinho, C. Amelung, D. Amidei, S. P. Amor Dos Santos, S. Amoroso, G. Amundsen, C. Anastopoulos, L. S. Ancu, N. Andari, T. Andeen, C. F. Anders, J. K. Anders, K. J. Anderson, A. Andreazza, V. Andrei, S. Angelidakis, I. Angelozzi, A. Angerami, A. V. Anisenkov, N. Anjos, A. Annovi, C. Antel, M. Antonelli, A. Antonov, D. J. Antrim, F. Anulli, M. Aoki, L. Aperio Bella, G. Arabidze, Y. Arai, J. P. Araque, V. Araujo Ferraz, A. T. H. Arce, R. E. Ardell, F. A. Arduh, J-F. Arguin, S. Argyropoulos, M. Arik, A. J. Armbruster, L. J. Armitage, O. Arnaez, H. Arnold, M. Arratia, O. Arslan, A. Artamonov, G. Artoni, S. Artz, S. Asai, N. Asbah, A. Ashkenazi, L. Asquith, K. Assamagan, R. Astalos, M. Atkinson, N. B. Atlay, K. Augsten, G. Avolio, B. Axen, M. K. Ayoub, G. Azuelos, A. E. Baas, M. J. Baca, H. Bachacou, K. Bachas, M. Backes, P. Bagnaia, M. Bahmani, H. Bahrasemani, J. T. Baines, M. Bajic, O. K. Baker, P. J. Bakker, E. M. Baldin, P. Balek, F. Balli, W. K. Balunas, E. Banas, A. Bandyopadhyay, Sw. Banerjee, A. A. E. Bannoura, L. Barak, E. L. Barberio, D. Barberis, M. Barbero, T. Barillari, M-S Barisits, J. T. Barkeloo, T. Barklow, N. Barlow, S. L. Barnes, B. M. Barnett, R. M. Barnett, Z. Barnovska-Blenessy, A. Baroncelli, G. Barone, A. J. Barr, L. Barranco Navarro, F. Barreiro, J. Barreiro Guimarães da Costa, R. Bartoldus, A. E. Barton, P. Bartos, A. Basalaev, A. Bassalat, R. L. Bates, S. J. Batista, J. R. Batley, M. Battaglia, M. Bauce, F. Bauer, H. S. Bawa, J. B. Beacham, M. D. Beattie, T. Beau, P. H. Beauchemin, P. Bechtle, H. P. Beck, H. C. Beck, K. Becker, M. Becker, C. Becot, A. J. Beddall, A. Beddall, V. A. Bednyakov, M. Bedognetti, C. P. Bee, T. A. Beermann, M. Begalli, M. Begel, J. K. Behr, A. S. Bell, G. Bella, L. Bellagamba, A. Bellerive, M. Bellomo, K. Belotskiy, O. Beltramello, N. L. Belyaev, O. Benary, D. Benchekroun, M. Bender, N. Benekos, Y. Benhammou, E. Benhar Noccioli, J. Benitez, D. P. Benjamin, M. Benoit, J. R. Bensinger, S. Bentvelsen, L. Beresford, M. Beretta, D. Berge, E. Bergeaas Kuutmann, N. Berger, L. J. Bergsten, J. Beringer, S. Berlendis, N. R. Bernard, G. Bernardi, C. Bernius, F. U. Bernlochner, T. Berry, P. Berta, C. Bertella, G. Bertoli, I. A. Bertram, C. Bertsche, G. J. Besjes, O. Bessidskaia Bylund, M. Bessner, N. Besson, A. Bethani, S. Bethke, A. Betti, A. J. Bevan, J. Beyer, R. M. Bianchi, O. Biebel, D. Biedermann, R. Bielski, K. Bierwagen, N. V. Biesuz, M. Biglietti, T. R. V. Billoud, H. Bilokon, M. Bindi, A. Bingul, C. Bini, S. Biondi, T. Bisanz, C. Bittrich, D. M. Bjergaard, J. E. Black, K. M. Black, R. E. Blair, T. Blazek, I. Bloch, C. Blocker, A. Blue, U. Blumenschein, Dr. Blunier, G. J. Bobbink, V. S. Bobrovnikov, S. S. Bocchetta, A. Bocci, C. Bock, M. Boehler, D. Boerner, D. Bogavac, A. G. Bogdanchikov, C. Bohm, V. Boisvert, P. Bokan, T. Bold, A. S. Boldyrev, A. E. Bolz, M. Bomben, M. Bona, M. Boonekamp, A. Borisov, G. Borissov, J. Bortfeldt, D. Bortoletto, V. Bortolotto, D. Boscherini, M. Bosman, J. D. Bossio Sola, J. Boudreau, E. V. Bouhova-Thacker, D. Boumediene, C. Bourdarios, S. K. Boutle, A. Boveia, J. Boyd, I. R. Boyko, A. J. Bozson, J. Bracinik, A. Brandt, G. Brandt, O. Brandt, F. Braren, U. Bratzler, B. Brau, J. E. Brau, W. D. Breaden Madden, K. Brendlinger, A. J. Brennan, L. Brenner, R. Brenner, S. Bressler, D. L. Briglin, T. M. Bristow, D. Britton, D. Britzger, F. M. Brochu, I. Brock, R. Brock, G. Brooijmans, T. Brooks, W. K. Brooks, J. Brosamer, E. Brost, J. H Broughton, P. A. Bruckman de Renstrom, D. Bruncko, A. Bruni, G. Bruni, L. S. Bruni, S. Bruno, BH Brunt, M. Bruschi, N. Bruscino, P. Bryant, L. Bryngemark, T. Buanes, Q. Buat, P. Buchholz, A. G. Buckley, I. A. Budagov, F. Buehrer, M. K. Bugge, O. Bulekov, D. Bullock, T. J. Burch, S. Burdin, C. D. Burgard, A. M. Burger, B. Burghgrave, K. Burka, S. Burke, I. Burmeister, J. T. P. Burr, D. Büscher, V. Büscher, P. Bussey, J. M. Butler, C. M. Buttar, J. M. Butterworth, P. Butti, W. Buttinger, A. Buzatu, A. R. Buzykaev, S. Cabrera Urbán, D. Caforio, H. Cai, V. M. Cairo, O. Cakir, N. Calace, P. Calafiura, A. Calandri, G. Calderini, P. Calfayan, G. Callea, L. P. Caloba, S. Calvente Lopez, D. Calvet, S. Calvet, T. P. Calvet, R. Camacho Toro, S. Camarda, P. Camarri, D. Cameron, R. Caminal Armadans, C. Camincher, S. Campana, M. Campanelli, A. Camplani, A. Campoverde, V. Canale, M. Cano Bret, J. Cantero, T. Cao, M. D. M. Capeans Garrido, I. Caprini, M. Caprini, M. Capua, R. M. Carbone, R. Cardarelli, F. Cardillo, I. Carli, T. Carli, G. Carlino, B. T. Carlson, L. Carminati, R. M. D. Carney, S. Caron, E. Carquin, S. Carrá, G. D. Carrillo-Montoya, D. Casadei, M. P. Casado, A. F. Casha, M. Casolino, D. W. Casper, R. Castelijn, V. Castillo Gimenez, N. F. Castro, A. Catinaccio, J. R. Catmore, A. Cattai, J. Caudron, V. Cavaliere, E. Cavallaro, D. Cavalli, M. Cavalli-Sforza, V. Cavasinni, E. Celebi, F. Ceradini, L. Cerda Alberich, A. S. Cerqueira, A. Cerri, L. Cerrito, F. Cerutti, A. Cervelli, S. A. Cetin, A. Chafaq, D. Chakraborty, S. K. Chan, W. S. Chan, Y. L. Chan, P. Chang, J. D. Chapman, D. G. Charlton, C. C. Chau, C. A. Chavez Barajas, S. Che, S. Cheatham, A. Chegwidden, S. Chekanov, S. V. Chekulaev, G. A. Chelkov, M. A. Chelstowska, C. Chen, C. Chen, H. Chen, J. Chen, S. Chen, S. Chen, X. Chen, Y. Chen, H. C. Cheng, H. J. Cheng, A. Cheplakov, E. Cheremushkina, R. Cherkaoui El Moursli, E. Cheu, K. Cheung, L. Chevalier, V. Chiarella, G. Chiarelli, G. Chiodini, A. S. Chisholm, A. Chitan, Y. H. Chiu, M. V. Chizhov, K. Choi, A. R. Chomont, S. Chouridou, Y. S. Chow, V. Christodoulou, M. C. Chu, J. Chudoba, A. J. Chuinard, J. J. Chwastowski, L. Chytka, A. K. Ciftci, D. Cinca, V. Cindro, I. A. Cioară, A. Ciocio, F. Cirotto, Z. H. Citron, M. Citterio, M. Ciubancan, A. Clark, B. L. Clark, M. R. Clark, P. J. Clark, R. N. Clarke, C. Clement, Y. Coadou, M. Cobal, A. Coccaro, J. Cochran, L. Colasurdo, B. Cole, A. P. Colijn, J. Collot, T. Colombo, P. Conde Muiño, E. Coniavitis, S. H. Connell, I. A. Connelly, S. Constantinescu, G. Conti, F. Conventi, M. Cooke, A. M. Cooper-Sarkar, F. Cormier, K. J. R. Cormier, M. Corradi, F. Corriveau, A. Cortes-Gonzalez, G. Costa, M. J. Costa, D. Costanzo, G. Cottin, G. Cowan, B. E. Cox, K. Cranmer, S. J. Crawley, R. A. Creager, G. Cree, S. Crépé-Renaudin, F. Crescioli, W. A. Cribbs, M. Cristinziani, V. Croft, G. Crosetti, A. Cueto, T. Cuhadar Donszelmann, A. R. Cukierman, J. Cummings, M. Curatolo, J. Cúth, S. Czekierda, P. Czodrowski, G. D’amen, S. D’Auria, L. D’eramo, M. D’Onofrio, M. J. Da Cunha Sargedas De Sousa, C. Da Via, W. Dabrowski, T. Dado, T. Dai, O. Dale, F. Dallaire, C. Dallapiccola, M. Dam, J. R. Dandoy, M. F. Daneri, N. P. Dang, A. C. Daniells, N. S. Dann, M. Danninger, M. Dano Hoffmann, V. Dao, G. Darbo, S. Darmora, J. Dassoulas, A. Dattagupta, T. Daubney, W. Davey, C. David, T. Davidek, D. R. Davis, P. Davison, E. Dawe, I. Dawson, K. De, R. de Asmundis, A. De Benedetti, S. De Castro, S. De Cecco, N. De Groot, P. de Jong, H. De la Torre, F. De Lorenzi, A. De Maria, D. De Pedis, A. De Salvo, U. De Sanctis, A. De Santo, K. De Vasconcelos Corga, J. B. De Vivie De Regie, R. Debbe, C. Debenedetti, D. V. Dedovich, N. Dehghanian, I. Deigaard, M. Del Gaudio, J. Del Peso, D. Delgove, F. Deliot, C. M. Delitzsch, A. Dell’Acqua, L. Dell’Asta, M. Dell’Orso, M. Della Pietra, D. della Volpe, M. Delmastro, C. Delporte, P. A. Delsart, D. A. DeMarco, S. Demers, M. Demichev, A. Demilly, S. P. Denisov, D. Denysiuk, D. Derendarz, J. E. Derkaoui, F. Derue, P. Dervan, K. Desch, C. Deterre, K. Dette, M. R. Devesa, P. O. Deviveiros, A. Dewhurst, S. Dhaliwal, F. A. Di Bello, A. Di Ciaccio, L. Di Ciaccio, W. K. Di Clemente, C. Di Donato, A. Di Girolamo, B. Di Girolamo, B. Di Micco, R. Di Nardo, K. F. Di Petrillo, A. Di Simone, R. Di Sipio, D. Di Valentino, C. Diaconu, M. Diamond, F. A. Dias, M. A. Diaz, J. Dickinson, E. B. Diehl, J. Dietrich, S. Díez Cornell, A. Dimitrievska, J. Dingfelder, P. Dita, S. Dita, F. Dittus, F. Djama, T. Djobava, J. I. Djuvsland, M. A. B. do Vale, D. Dobos, M. Dobre, D. Dodsworth, C. Doglioni, J. Dolejsi, Z. Dolezal, M. Donadelli, S. Donati, P. Dondero, J. Donini, J. Dopke, A. Doria, M. T. Dova, A. T. Doyle, E. Drechsler, M. Dris, Y. Du, J. Duarte-Campderros, F. Dubinin, A. Dubreuil, E. Duchovni, G. Duckeck, A. Ducourthial, O. A. Ducu, D. Duda, A. Dudarev, A. Chr. Dudder, E. M. Duffield, L. Duflot, M. Dührssen, C. Dulsen, M. Dumancic, A. E. Dumitriu, A. K. Duncan, M. Dunford, A. Duperrin, H. Duran Yildiz, M. Düren, A. Durglishvili, D. Duschinger, B. Dutta, D. Duvnjak, M. Dyndal, B. S. Dziedzic, C. Eckardt, K. M. Ecker, R. C. Edgar, T. Eifert, G. Eigen, K. Einsweiler, T. Ekelof, M. El Kacimi, R. El Kosseifi, V. Ellajosyula, M. Ellert, S. Elles, F. Ellinghaus, A. A. Elliot, N. Ellis, J. Elmsheuser, M. Elsing, D. Emeliyanov, Y. Enari, J. S. Ennis, M. B. Epland, J. Erdmann, A. Ereditato, M. Ernst, S. Errede, M. Escalier, C. Escobar, B. Esposito, O. Estrada Pastor, A. I. Etienvre, E. Etzion, H. Evans, A. Ezhilov, M. Ezzi, F. Fabbri, L. Fabbri, V. Fabiani, G. Facini, R. M. Fakhrutdinov, S. Falciano, R. J. Falla, J. Faltova, Y. Fang, M. Fanti, A. Farbin, A. Farilla, C. Farina, E. M. Farina, T. Farooque, S. Farrell, S. M. Farrington, P. Farthouat, F. Fassi, P. Fassnacht, D. Fassouliotis, M. Faucci Giannelli, A. Favareto, W. J. Fawcett, L. Fayard, O. L. Fedin, W. Fedorko, S. Feigl, L. Feligioni, C. Feng, E. J. Feng, M. J. Fenton, A. B. Fenyuk, L. Feremenga, P. Fernandez Martinez, J. Ferrando, A. Ferrari, P. Ferrari, R. Ferrari, D. E. Ferreira de Lima, A. Ferrer, D. Ferrere, C. Ferretti, F. Fiedler, A. Filipčič, M. Filipuzzi, F. Filthaut, M. Fincke-Keeler, K. D. Finelli, M. C. N. Fiolhais, L. Fiorini, A. Fischer, C. Fischer, J. Fischer, W. C. Fisher, N. Flaschel, I. Fleck, P. Fleischmann, R. R. M. Fletcher, T. Flick, B. M. Flierl, L. R. Flores Castillo, M. J. Flowerdew, G. T. Forcolin, A. Formica, F. A. Förster, A. Forti, A. G. Foster, D. Fournier, H. Fox, S. Fracchia, P. Francavilla, M. Franchini, S. Franchino, D. Francis, L. Franconi, M. Franklin, M. Frate, M. Fraternali, D. Freeborn, S. M. Fressard-Batraneanu, B. Freund, D. Froidevaux, J. A. Frost, C. Fukunaga, T. Fusayasu, J. Fuster, O. Gabizon, A. Gabrielli, A. Gabrielli, G. P. Gach, S. Gadatsch, S. Gadomski, G. Gagliardi, L. G. Gagnon, C. Galea, B. Galhardo, E. J. Gallas, B. J. Gallop, P. Gallus, G. Galster, K. K. Gan, S. Ganguly, Y. Gao, Y. S. Gao, F. M. Garay Walls, C. García, J. E. García Navarro, J. A. García Pascual, M. Garcia-Sciveres, R. W. Gardner, N. Garelli, V. Garonne, A. Gascon Bravo, K. Gasnikova, C. Gatti, A. Gaudiello, G. Gaudio, I. L. Gavrilenko, C. Gay, G. Gaycken, E. N. Gazis, C. N. P. Gee, J. Geisen, M. Geisen, M. P. Geisler, K. Gellerstedt, C. Gemme, M. H. Genest, C. Geng, S. Gentile, C. Gentsos, S. George, D. Gerbaudo, G. Geßner, S. Ghasemi, M. Ghneimat, B. Giacobbe, S. Giagu, N. Giangiacomi, P. Giannetti, S. M. Gibson, M. Gignac, M. Gilchriese, D. Gillberg, G. Gilles, D. M. Gingrich, M. P. Giordani, F. M. Giorgi, P. F. Giraud, P. Giromini, G. Giugliarelli, D. Giugni, F. Giuli, C. Giuliani, M. Giulini, B. K. Gjelsten, S. Gkaitatzis, I. Gkialas, E. L. Gkougkousis, P. Gkountoumis, L. K. Gladilin, C. Glasman, J. Glatzer, P. C. F. Glaysher, A. Glazov, M. Goblirsch-Kolb, J. Godlewski, S. Goldfarb, T. Golling, D. Golubkov, A. Gomes, R. Gonçalo, R. Goncalves Gama, J. Goncalves Pinto Firmino Da Costa, G. Gonella, L. Gonella, A. Gongadze, J. L. Gonski, S. González de la Hoz, S. Gonzalez-Sevilla, L. Goossens, P. A. Gorbounov, H. A. Gordon, I. Gorelov, B. Gorini, E. Gorini, A. Gorišek, A. T. Goshaw, C. Gössling, M. I. Gostkin, C. A. Gottardo, C. R. Goudet, D. Goujdami, A. G. Goussiou, N. Govender, E. Gozani, I. Grabowska-Bold, P. O. J. Gradin, J. Gramling, E. Gramstad, S. Grancagnolo, V. Gratchev, P. M. Gravila, C. Gray, H. M. Gray, Z. D. Greenwood, C. Grefe, K. Gregersen, I. M. Gregor, P. Grenier, K. Grevtsov, J. Griffiths, A. A. Grillo, K. Grimm, S. Grinstein, Ph. Gris, J.-F. Grivaz, S. Groh, E. Gross, J. Grosse-Knetter, G. C. Grossi, Z. J. Grout, A. Grummer, L. Guan, W. Guan, J. Guenther, F. Guescini, D. Guest, O. Gueta, B. Gui, E. Guido, T. Guillemin, S. Guindon, U. Gul, C. Gumpert, J. Guo, W. Guo, Y. Guo, R. Gupta, S. Gurbuz, G. Gustavino, B. J. Gutelman, P. Gutierrez, N. G. Gutierrez Ortiz, C. Gutschow, C. Guyot, M. P. Guzik, C. Gwenlan, C. B. Gwilliam, A. Haas, C. Haber, H. K. Hadavand, N. Haddad, A. Hadef, S. Hageböck, M. Hagihara, H. Hakobyan, M. Haleem, J. Haley, G. Halladjian, G. D. Hallewell, K. Hamacher, P. Hamal, K. Hamano, A. Hamilton, G. N. Hamity, P. G. Hamnett, L. Han, S. Han, K. Hanagaki, K. Hanawa, M. Hance, D. M. Handl, B. Haney, P. Hanke, J. B. Hansen, J. D. Hansen, M. C. Hansen, P. H. Hansen, K. Hara, A. S. Hard, T. Harenberg, F. Hariri, S. Harkusha, P. F. Harrison, N. M. Hartmann, Y. Hasegawa, A. Hasib, S. Hassani, S. Haug, R. Hauser, L. Hauswald, L. B. Havener, M. Havranek, C. M. Hawkes, R. J. Hawkings, D. Hayakawa, D. Hayden, C. P. Hays, J. M. Hays, H. S. Hayward, S. J. Haywood, S. J. Head, T. Heck, V. Hedberg, L. Heelan, S. Heer, K. K. Heidegger, S. Heim, T. Heim, B. Heinemann, J. J. Heinrich, L. Heinrich, C. Heinz, J. Hejbal, L. Helary, A. Held, S. Hellman, C. Helsens, R. C. W. Henderson, Y. Heng, S. Henkelmann, A. M. Henriques Correia, S. Henrot-Versille, G. H. Herbert, H. Herde, V. Herget, Y. Hernández Jiménez, H. Herr, G. Herten, R. Hertenberger, L. Hervas, T. C. Herwig, G. G. Hesketh, N. P. Hessey, J. W. Hetherly, S. Higashino, E. Higón-Rodriguez, K. Hildebrand, E. Hill, J. C. Hill, K. H. Hiller, S. J. Hillier, M. Hils, I. Hinchliffe, M. Hirose, D. Hirschbuehl, B. Hiti, O. Hladik, D. R. Hlaluku, X. Hoad, J. Hobbs, N. Hod, M. C. Hodgkinson, P. Hodgson, A. Hoecker, M. R. Hoeferkamp, F. Hoenig, D. Hohn, T. R. Holmes, M. Holzbock, M. Homann, S. Honda, T. Honda, T. M. Hong, B. H. Hooberman, W. H. Hopkins, Y. Horii, A. J. Horton, J-Y. Hostachy, A. Hostiuc, S. Hou, A. Hoummada, J. Howarth, J. Hoya, M. Hrabovsky, J. Hrdinka, I. Hristova, J. Hrivnac, T. Hryn’ova, A. Hrynevich, P. J. Hsu, S.-C. Hsu, Q. Hu, S. Hu, Y. Huang, Z. Hubacek, F. Hubaut, F. Huegging, T. B. Huffman, E. W. Hughes, M. Huhtinen, R. F. H. Hunter, P. Huo, N. Huseynov, J. Huston, J. Huth, R. Hyneman, G. Iacobucci, G. Iakovidis, I. Ibragimov, L. Iconomidou-Fayard, Z. Idrissi, P. Iengo, O. Igonkina, T. Iizawa, Y. Ikegami, M. Ikeno, Y. Ilchenko, D. Iliadis, N. Ilic, F. Iltzsche, G. Introzzi, P. Ioannou, M. Iodice, K. Iordanidou, V. Ippolito, M. F. Isacson, N. Ishijima, M. Ishino, M. Ishitsuka, C. Issever, S. Istin, F. Ito, J. M. Iturbe Ponce, R. Iuppa, H. Iwasaki, J. M. Izen, V. Izzo, S. Jabbar, P. Jackson, R. M. Jacobs, V. Jain, K. B. Jakobi, K. Jakobs, S. Jakobsen, T. Jakoubek, D. O. Jamin, D. K. Jana, R. Jansky, J. Janssen, M. Janus, P. A. Janus, G. Jarlskog, N. Javadov, T. Javůrek, M. Javurkova, F. Jeanneau, L. Jeanty, J. Jejelava, A. Jelinskas, P. Jenni, C. Jeske, S. Jézéquel, H. Ji, J. Jia, H. Jiang, Y. Jiang, Z. Jiang, S. Jiggins, J. Jimenez Pena, S. Jin, A. Jinaru, O. Jinnouchi, H. Jivan, P. Johansson, K. A. Johns, C. A. Johnson, W. J. Johnson, K. Jon-And, R. W. L. Jones, S. D. Jones, S. Jones, T. J. Jones, J. Jongmanns, P. M. Jorge, J. Jovicevic, X. Ju, A. Juste Rozas, M. K. Köhler, A. Kaczmarska, M. Kado, H. Kagan, M. Kagan, S. J. Kahn, T. Kaji, E. Kajomovitz, C. W. Kalderon, A. Kaluza, S. Kama, A. Kamenshchikov, N. Kanaya, L. Kanjir, V. A. Kantserov, J. Kanzaki, B. Kaplan, L. S. Kaplan, D. Kar, K. Karakostas, N. Karastathis, M. J. Kareem, E. Karentzos, S. N. Karpov, Z. M. Karpova, K. Karthik, V. Kartvelishvili, A. N. Karyukhin, K. Kasahara, L. Kashif, R. D. Kass, A. Kastanas, Y. Kataoka, C. Kato, A. Katre, J. Katzy, K. Kawade, K. Kawagoe, T. Kawamoto, G. Kawamura, E. F. Kay, V. F. Kazanin, R. Keeler, R. Kehoe, J. S. Keller, E. Kellermann, J. J. Kempster, J Kendrick, H. Keoshkerian, O. Kepka, B. P. Kerševan, S. Kersten, R. A. Keyes, M. Khader, F. Khalil-zada, A. Khanov, A. G. Kharlamov, T. Kharlamova, A. Khodinov, T. J. Khoo, V. Khovanskiy, E. Khramov, J. Khubua, S. Kido, C. R. Kilby, H. Y. Kim, S. H. Kim, Y. K. Kim, N. Kimura, O. M. Kind, B. T. King, D. Kirchmeier, J. Kirk, A. E. Kiryunin, T. Kishimoto, D. Kisielewska, V. Kitali, O. Kivernyk, E. Kladiva, T. Klapdor-Kleingrothaus, M. H. Klein, M. Klein, U. Klein, K. Kleinknecht, P. Klimek, A. Klimentov, R. Klingenberg, T. Klingl, T. Klioutchnikova, F. F. Klitzner, E.-E. Kluge, P. Kluit, S. Kluth, E. Kneringer, E. B. F. G. Knoops, A. Knue, A. Kobayashi, D. Kobayashi, T. Kobayashi, M. Kobel, M. Kocian, P. Kodys, T. Koffas, E. Koffeman, N. M. Köhler, T. Koi, M. Kolb, I. Koletsou, A. A. Komar, T. Kondo, N. Kondrashova, K. Köneke, A. C. König, T. Kono, R. Konoplich, N. Konstantinidis, B. Konya, R. Kopeliansky, S. Koperny, A. K. Kopp, K. Korcyl, K. Kordas, A. Korn, A. A. Korol, I. Korolkov, E. V. Korolkova, O. Kortner, S. Kortner, T. Kosek, V. V. Kostyukhin, A. Kotwal, A. Koulouris, A. Kourkoumeli-Charalampidi, C. Kourkoumelis, E. Kourlitis, V. Kouskoura, A. B. Kowalewska, R. Kowalewski, T. Z. Kowalski, C. Kozakai, W. Kozanecki, A. S. Kozhin, V. A. Kramarenko, G. Kramberger, D. Krasnopevtsev, M. W. Krasny, A. Krasznahorkay, D. Krauss, J. A. Kremer, J. Kretzschmar, K. Kreutzfeldt, P. Krieger, K. Krizka, K. Kroeninger, H. Kroha, J. Kroll, J. Kroll, J. Kroseberg, J. Krstic, U. Kruchonak, H. Krüger, N. Krumnack, M. C. Kruse, T. Kubota, H. Kucuk, S. Kuday, J. T. Kuechler, S. Kuehn, A. Kugel, F. Kuger, T. Kuhl, V. Kukhtin, R. Kukla, Y. Kulchitsky, S. Kuleshov, Y. P. Kulinich, M. Kuna, T. Kunigo, A. Kupco, T. Kupfer, O. Kuprash, H. Kurashige, L. L. Kurchaninov, Y. A. Kurochkin, M. G. Kurth, E. S. Kuwertz, M. Kuze, J. Kvita, T. Kwan, D. Kyriazopoulos, A. La Rosa, J. L. La Rosa Navarro, L. La Rotonda, F. La Ruffa, C. Lacasta, F. Lacava, J. Lacey, D. P. J. Lack, H. Lacker, D. Lacour, E. Ladygin, R. Lafaye, B. Laforge, S. Lai, S. Lammers, W. Lampl, E. Lançon, U. Landgraf, M. P. J. Landon, M. C. Lanfermann, V. S. Lang, J. C. Lange, R. J. Langenberg, A. J. Lankford, F. Lanni, K. Lantzsch, A. Lanza, A. Lapertosa, S. Laplace, J. F. Laporte, T. Lari, F. Lasagni Manghi, M. Lassnig, T. S. Lau, P. Laurelli, W. Lavrijsen, A. T. Law, P. Laycock, T. Lazovich, M. Lazzaroni, B. Le, O. Le Dortz, E. Le Guirriec, E. P. Le Quilleuc, M. LeBlanc, T. LeCompte, F. Ledroit-Guillon, C. A. Lee, G. R. Lee, S. C. Lee, L. Lee, B. Lefebvre, G. Lefebvre, M. Lefebvre, F. Legger, C. Leggett, G. Lehmann Miotto, X. Lei, W. A. Leight, M. A. L. Leite, R. Leitner, D. Lellouch, B. Lemmer, K. J. C. Leney, T. Lenz, B. Lenzi, R. Leone, S. Leone, C. Leonidopoulos, G. Lerner, C. Leroy, R. Les, A. A. J. Lesage, C. G. Lester, M. Levchenko, J. Levêque, D. Levin, L. J. Levinson, M. Levy, D. Lewis, B. Li, C.-Q. Li, H. Li, L. Li, Q. Li, S. Li, X. Li, Y. Li, Z. Liang, B. Liberti, A. Liblong, K. Lie, J. Liebal, W. Liebig, A. Limosani, C. Y. Lin, K. Lin, S. C. Lin, T. H. Lin, R. A. Linck, B. E. Lindquist, A. E. Lionti, E. Lipeles, A. Lipniacka, M. Lisovyi, T. M. Liss, A. Lister, A. M. Litke, B. Liu, H. Liu, H. Liu, J. K. K. Liu, J. Liu, J. B. Liu, K. Liu, L. Liu, M. Liu, Y. L. Liu, Y. Liu, M. Livan, A. Lleres, J. Llorente Merino, S. L. Lloyd, C. Y. Lo, F. Lo Sterzo, E. M. Lobodzinska, P. Loch, F. K. Loebinger, A. Loesle, K. M. Loew, T. Lohse, K. Lohwasser, M. Lokajicek, B. A. Long, J. D. Long, R. E. Long, L. Longo, K. A. Looper, J. A. Lopez, I. Lopez Paz, A. Lopez Solis, J. Lorenz, N. Lorenzo Martinez, M. Losada, P. J. Lösel, X. Lou, A. Lounis, J. Love, P. A. Love, H. Lu, N. Lu, Y. J. Lu, H. J. Lubatti, C. Luci, A. Lucotte, C. Luedtke, F. Luehring, W. Lukas, L. Luminari, O. Lundberg, B. Lund-Jensen, M. S. Lutz, P. M. Luzi, D. Lynn, R. Lysak, E. Lytken, F. Lyu, V. Lyubushkin, H. Ma, L. L. Ma, Y. Ma, G. Maccarrone, A. Macchiolo, C. M. Macdonald, B. Maček, J. Machado Miguens, D. Madaffari, R. Madar, W. F. Mader, A. Madsen, N. Madysa, J. Maeda, S. Maeland, T. Maeno, A. S. Maevskiy, V. Magerl, C. Maiani, C. Maidantchik, T. Maier, A. Maio, O. Majersky, S. Majewski, Y. Makida, N. Makovec, B. Malaescu, Pa. Malecki, V. P. Maleev, F. Malek, U. Mallik, D. Malon, C. Malone, S. Maltezos, S. Malyukov, J. Mamuzic, G. Mancini, I. Mandić, J. Maneira, L. Manhaes de Andrade Filho, J. Manjarres Ramos, K. H. Mankinen, A. Mann, A. Manousos, B. Mansoulie, J. D. Mansour, R. Mantifel, M. Mantoani, S. Manzoni, L. Mapelli, G. Marceca, L. March, L. Marchese, G. Marchiori, M. Marcisovsky, C. A. Marin Tobon, M. Marjanovic, D. E. Marley, F. Marroquim, S. P. Marsden, Z. Marshall, M. U. F Martensson, S. Marti-Garcia, C. B. Martin, T. A. Martin, V. J. Martin, B. Martin dit Latour, M. Martinez, V. I. Martinez Outschoorn, S. Martin-Haugh, V. S. Martoiu, A. C. Martyniuk, A. Marzin, L. Masetti, T. Mashimo, R. Mashinistov, J. Masik, A. L. Maslennikov, L. H. Mason, L. Massa, P. Mastrandrea, A. Mastroberardino, T. Masubuchi, P. Mättig, J. Maurer, S. J. Maxfield, D. A. Maximov, R. Mazini, I. Maznas, S. M. Mazza, N. C. Mc Fadden, G. Mc Goldrick, S. P. Mc Kee, A. McCarn, R. L. McCarthy, T. G. McCarthy, L. I. McClymont, E. F. McDonald, J. A. Mcfayden, G. Mchedlidze, S. J. McMahon, P. C. McNamara, C. J. McNicol, R. A. McPherson, S. Meehan, T. J. Megy, S. Mehlhase, A. Mehta, T. Meideck, K. Meier, B. Meirose, D. Melini, B. R. Mellado Garcia, J. D. Mellenthin, M. Melo, F. Meloni, A. Melzer, S. B. Menary, L. Meng, X. T. Meng, A. Mengarelli, S. Menke, E. Meoni, S. Mergelmeyer, C. Merlassino, P. Mermod, L. Merola, C. Meroni, F. S. Merritt, A. Messina, J. Metcalfe, A. S. Mete, C. Meyer, J-P. Meyer, J. Meyer, H. Meyer Zu Theenhausen, F. Miano, R. P. Middleton, S. Miglioranzi, L. Mijović, G. Mikenberg, M. Mikestikova, M. Mikuž, M. Milesi, A. Milic, D. A. Millar, D. W. Miller, C. Mills, A. Milov, D. A. Milstead, A. A. Minaenko, Y. Minami, I. A. Minashvili, A. I. Mincer, B. Mindur, M. Mineev, Y. Minegishi, Y. Ming, L. M. Mir, A. Mirto, K. P. Mistry, T. Mitani, J. Mitrevski, V. A. Mitsou, A. Miucci, P. S. Miyagawa, A. Mizukami, J. U. Mjörnmark, T. Mkrtchyan, M. Mlynarikova, T. Moa, K. Mochizuki, P. Mogg, S. Mohapatra, S. Molander, R. Moles-Valls, M. C. Mondragon, K. Mönig, J. Monk, E. Monnier, A. Montalbano, J. Montejo Berlingen, F. Monticelli, S. Monzani, R. W. Moore, N. Morange, D. Moreno, M. Moreno Llácer, P. Morettini, S. Morgenstern, D. Mori, T. Mori, M. Morii, M. Morinaga, V. Morisbak, A. K. Morley, G. Mornacchi, J. D. Morris, L. Morvaj, P. Moschovakos, M. Mosidze, H. J. Moss, J. Moss, K. Motohashi, R. Mount, E. Mountricha, E. J. W. Moyse, S. Muanza, F. Mueller, J. Mueller, R. S. P. Mueller, D. Muenstermann, P. Mullen, G. A. Mullier, F. J. Munoz Sanchez, W. J. Murray, H. Musheghyan, M. Muškinja, A. G. Myagkov, M. Myska, B. P. Nachman, O. Nackenhorst, K. Nagai, R. Nagai, K. Nagano, Y. Nagasaka, K. Nagata, M. Nagel, E. Nagy, A. M. Nairz, Y. Nakahama, K. Nakamura, T. Nakamura, I. Nakano, R. F. Naranjo Garcia, R. Narayan, D. I. Narrias Villar, I. Naryshkin, T. Naumann, G. Navarro, R. Nayyar, H. A. Neal, P. Yu. Nechaeva, T. J. Neep, A. Negri, M. Negrini, S. Nektarijevic, C. Nellist, A. Nelson, M. E. Nelson, S. Nemecek, P. Nemethy, M. Nessi, M. S. Neubauer, M. Neumann, P. R. Newman, T. Y. Ng, Y. S. Ng, T. Nguyen Manh, R. B. Nickerson, R. Nicolaidou, J. Nielsen, N. Nikiforou, V. Nikolaenko, I. Nikolic-Audit, K. Nikolopoulos, P. Nilsson, Y. Ninomiya, A. Nisati, N. Nishu, R. Nisius, I. Nitsche, T. Nitta, T. Nobe, Y. Noguchi, M. Nomachi, I. Nomidis, M. A. Nomura, T. Nooney, M. Nordberg, N. Norjoharuddeen, O. Novgorodova, M. Nozaki, L. Nozka, K. Ntekas, E. Nurse, F. Nuti, K. O’connor, D. C. O’Neil, A. A. O’Rourke, V. O’Shea, F. G. Oakham, H. Oberlack, T. Obermann, J. Ocariz, A. Ochi, I. Ochoa, J. P. Ochoa-Ricoux, S. Oda, S. Odaka, A. Oh, S. H. Oh, C. C. Ohm, H. Ohman, H. Oide, H. Okawa, Y. Okumura, T. Okuyama, A. Olariu, L. F. Oleiro Seabra, S. A. Olivares Pino, D. Oliveira Damazio, M. J. R. Olsson, A. Olszewski, J. Olszowska, A. Onofre, K. Onogi, P. U. E. Onyisi, H. Oppen, M. J. Oreglia, Y. Oren, D. Orestano, N. Orlando, R. S. Orr, B. Osculati, R. Ospanov, G. Otero y Garzon, H. Otono, M. Ouchrif, F. Ould-Saada, A. Ouraou, K. P. Oussoren, Q. Ouyang, M. Owen, R. E. Owen, V. E. Ozcan, N. Ozturk, K. Pachal, A. Pacheco Pages, L. Pacheco Rodriguez, C. Padilla Aranda, S. Pagan Griso, M. Paganini, F. Paige, G. Palacino, S. Palazzo, S. Palestini, M. Palka, D. Pallin, E. St. Panagiotopoulou, I. Panagoulias, C. E. Pandini, J. G. Panduro Vazquez, P. Pani, S. Panitkin, D. Pantea, L. Paolozzi, Th. D. Papadopoulou, K. Papageorgiou, A. Paramonov, D. Paredes Hernandez, A. J. Parker, M. A. Parker, K. A. Parker, F. Parodi, J. A. Parsons, U. Parzefall, V. R. Pascuzzi, J. M. Pasner, E. Pasqualucci, S. Passaggio, Fr. Pastore, S. Pataraia, J. R. Pater, T. Pauly, B. Pearson, S. Pedraza Lopez, R. Pedro, S. V. Peleganchuk, O. Penc, C. Peng, H. Peng, J. Penwell, B. S. Peralva, M. M. Perego, D. V. Perepelitsa, F. Peri, L. Perini, H. Pernegger, S. Perrella, R. Peschke, V. D. Peshekhonov, K. Peters, R. F. Y. Peters, B. A. Petersen, T. C. Petersen, E. Petit, A. Petridis, C. Petridou, P. Petroff, E. Petrolo, M. Petrov, F. Petrucci, N. E. Pettersson, A. Peyaud, R. Pezoa, F. H. Phillips, P. W. Phillips, G. Piacquadio, E. Pianori, A. Picazio, M. A. Pickering, R. Piegaia, J. E. Pilcher, A. D. Pilkington, M. Pinamonti, J. L. Pinfold, H. Pirumov, M. Pitt, L. Plazak, M.-A. Pleier, V. Pleskot, E. Plotnikova, D. Pluth, P. Podberezko, R. Poettgen, R. Poggi, L. Poggioli, I. Pogrebnyak, D. Pohl, I. Pokharel, G. Polesello, A. Poley, A. Policicchio, R. Polifka, A. Polini, C. S. Pollard, V. Polychronakos, K. Pommès, D. Ponomarenko, L. Pontecorvo, G. A. Popeneciu, D. M. Portillo Quintero, S. Pospisil, K. Potamianos, I. N. Potrap, C. J. Potter, H. Potti, T. Poulsen, J. Poveda, M. E. Pozo Astigarraga, P. Pralavorio, A. Pranko, S. Prell, D. Price, M. Primavera, S. Prince, N. Proklova, K. Prokofiev, F. Prokoshin, S. Protopopescu, J. Proudfoot, M. Przybycien, A. Puri, P. Puzo, J. Qian, G. Qin, Y. Qin, A. Quadt, M. Queitsch-Maitland, D. Quilty, S. Raddum, V. Radeka, V. Radescu, S. K. Radhakrishnan, P. Radloff, P. Rados, F. Ragusa, G. Rahal, J. A. Raine, S. Rajagopalan, C. Rangel-Smith, T. Rashid, S. Raspopov, M. G. Ratti, D. M. Rauch, F. Rauscher, S. Rave, I. Ravinovich, J. H. Rawling, M. Raymond, A. L. Read, N. P. Readioff, M. Reale, D. M. Rebuzzi, A. Redelbach, G. Redlinger, R. Reece, R. G. Reed, K. Reeves, L. Rehnisch, J. Reichert, A. Reiss, C. Rembser, H. Ren, M. Rescigno, S. Resconi, E. D. Resseguie, S. Rettie, E. Reynolds, O. L. Rezanova, P. Reznicek, R. Rezvani, R. Richter, S. Richter, E. Richter-Was, O. Ricken, M. Ridel, P. Rieck, C. J. Riegel, J. Rieger, O. Rifki, M. Rijssenbeek, A. Rimoldi, M. Rimoldi, L. Rinaldi, G. Ripellino, B. Ristić, E. Ritsch, I. Riu, F. Rizatdinova, E. Rizvi, C. Rizzi, R. T. Roberts, S. H. Robertson, A. Robichaud-Veronneau, D. Robinson, J. E. M. Robinson, A. Robson, E. Rocco, C. Roda, Y. Rodina, S. Rodriguez Bosca, A. Rodriguez Perez, D. Rodriguez Rodriguez, S. Roe, C. S. Rogan, O. Røhne, J. Roloff, A. Romaniouk, M. Romano, S. M. Romano Saez, E. Romero Adam, N. Rompotis, M. Ronzani, L. Roos, S. Rosati, K. Rosbach, P. Rose, N.-A. Rosien, E. Rossi, L. P. Rossi, J. H. N. Rosten, R. Rosten, M. Rotaru, J. Rothberg, D. Rousseau, D. Roy, A. Rozanov, Y. Rozen, X. Ruan, F. Rubbo, F. Rühr, A. Ruiz-Martinez, Z. Rurikova, N. A. Rusakovich, H. L. Russell, J. P. Rutherfoord, N. Ruthmann, E. M. Rüttinger, Y. F. Ryabov, M. Rybar, G. Rybkin, S. Ryu, A. Ryzhov, G. F. Rzehorz, A. F. Saavedra, G. Sabato, S. Sacerdoti, H. F.-W. Sadrozinski, R. Sadykov, F. Safai Tehrani, P. Saha, M. Sahinsoy, M. Saimpert, M. Saito, T. Saito, H. Sakamoto, Y. Sakurai, G. Salamanna, J. E. Salazar Loyola, D. Salek, P. H. Sales De Bruin, D. Salihagic, A. Salnikov, J. Salt, D. Salvatore, F. Salvatore, A. Salvucci, A. Salzburger, D. Sammel, D. Sampsonidis, D. Sampsonidou, J. Sánchez, V. Sanchez Martinez, A. Sanchez Pineda, H. Sandaker, R. L. Sandbach, C. O. Sander, M. Sandhoff, C. Sandoval, D. P. C. Sankey, M. Sannino, Y. Sano, A. Sansoni, C. Santoni, H. Santos, I. Santoyo Castillo, A. Sapronov, J. G. Saraiva, B. Sarrazin, O. Sasaki, K. Sato, E. Sauvan, G. Savage, P. Savard, N. Savic, C. Sawyer, L. Sawyer, J. Saxon, C. Sbarra, A. Sbrizzi, T. Scanlon, D. A. Scannicchio, J. Schaarschmidt, P. Schacht, B. M. Schachtner, D. Schaefer, L. Schaefer, R. Schaefer, J. Schaeffer, S. Schaepe, S. Schaetzel, U. Schäfer, A. C. Schaffer, D. Schaile, R. D. Schamberger, V. A. Schegelsky, D. Scheirich, F. Schenck, M. Schernau, C. Schiavi, S. Schier, L. K. Schildgen, C. Schillo, M. Schioppa, S. Schlenker, K. R. Schmidt-Sommerfeld, K. Schmieden, C. Schmitt, S. Schmitt, S. Schmitz, U. Schnoor, L. Schoeffel, A. Schoening, B. D. Schoenrock, E. Schopf, M. Schott, J. F. P. Schouwenberg, J. Schovancova, S. Schramm, N. Schuh, A. Schulte, M. J. Schultens, H.-C. Schultz-Coulon, H. Schulz, M. Schumacher, B. A. Schumm, Ph. Schune, A. Schwartzman, T. A. Schwarz, H. Schweiger, Ph. Schwemling, R. Schwienhorst, J. Schwindling, A. Sciandra, G. Sciolla, M. Scornajenghi, F. Scuri, F. Scutti, J. Searcy, P. Seema, S. C. Seidel, A. Seiden, J. M. Seixas, G. Sekhniaidze, K. Sekhon, S. J. Sekula, N. Semprini-Cesari, S. Senkin, C. Serfon, L. Serin, L. Serkin, M. Sessa, R. Seuster, H. Severini, T. Šfiligoj, F. Sforza, A. Sfyrla, E. Shabalina, N. W. Shaikh, L. Y. Shan, R. Shang, J. T. Shank, M. Shapiro, P. B. Shatalov, K. Shaw, S. M. Shaw, A. Shcherbakova, C. Y. Shehu, Y. Shen, N. Sherafati, A. D. Sherman, P. Sherwood, L. Shi, S. Shimizu, C. O. Shimmin, M. Shimojima, I. P. J. Shipsey, S. Shirabe, M. Shiyakova, J. Shlomi, A. Shmeleva, D. Shoaleh Saadi, M. J. Shochet, S. Shojaii, D. R. Shope, S. Shrestha, E. Shulga, M. A. Shupe, P. Sicho, A. M. Sickles, P. E. Sidebo, E. Sideras Haddad, O. Sidiropoulou, A. Sidoti, F. Siegert, Dj. Sijacki, J. Silva, S. B. Silverstein, V. Simak, L. Simic, S. Simion, E. Simioni, B. Simmons, M. Simon, P. Sinervo, N. B. Sinev, M. Sioli, G. Siragusa, I. Siral, S. Yu. Sivoklokov, J. Sjölin, M. B. Skinner, P. Skubic, M. Slater, T. Slavicek, M. Slawinska, K. Sliwa, R. Slovak, V. Smakhtin, B. H. Smart, J. Smiesko, N. Smirnov, S. Yu. Smirnov, Y. Smirnov, L. N. Smirnova, O. Smirnova, J. W. Smith, M. N. K. Smith, R. W. Smith, M. Smizanska, K. Smolek, A. A. Snesarev, I. M. Snyder, S. Snyder, R. Sobie, F. Socher, A. Soffer, A. Søgaard, D. A. Soh, G. Sokhrannyi, C. A. Solans Sanchez, M. Solar, E. Yu. Soldatov, U. Soldevila, A. A. Solodkov, A. Soloshenko, O. V. Solovyanov, V. Solovyev, P. Sommer, H. Son, A. Sopczak, D. Sosa, C. L. Sotiropoulou, S. Sottocornola, R. Soualah, A. M. Soukharev, D. South, B. C. Sowden, S. Spagnolo, M. Spalla, M. Spangenberg, F. Spanò, D. Sperlich, F. Spettel, T. M. Spieker, R. Spighi, G. Spigo, L. A. Spiller, M. Spousta, R. D. St. Denis, A. Stabile, R. Stamen, S. Stamm, E. Stanecka, R. W. Stanek, C. Stanescu, M. M. Stanitzki, B. S. Stapf, S. Stapnes, E. A. Starchenko, G. H. Stark, J. Stark, S. H Stark, P. Staroba, P. Starovoitov, S. Stärz, R. Staszewski, M. Stegler, P. Steinberg, B. Stelzer, H. J. Stelzer, O. Stelzer-Chilton, H. Stenzel, T. J. Stevenson, G. A. Stewart, M. C. Stockton, M. Stoebe, G. Stoicea, P. Stolte, S. Stonjek, A. R. Stradling, A. Straessner, M. E. Stramaglia, J. Strandberg, S. Strandberg, M. Strauss, P. Strizenec, R. Ströhmer, D. M. Strom, R. Stroynowski, A. Strubig, S. A. Stucci, B. Stugu, N. A. Styles, D. Su, J. Su, S. Suchek, Y. Sugaya, M. Suk, V. V. Sulin, DMS Sultan, S. Sultansoy, T. Sumida, S. Sun, X. Sun, K. Suruliz, C. J. E. Suster, M. R. Sutton, S. Suzuki, M. Svatos, M. Swiatlowski, S. P. Swift, I. Sykora, T. Sykora, D. Ta, K. Tackmann, J. Taenzer, A. Taffard, R. Tafirout, E. Tahirovic, N. Taiblum, H. Takai, R. Takashima, E. H. Takasugi, K. Takeda, T. Takeshita, Y. Takubo, M. Talby, A. A. Talyshev, J. Tanaka, M. Tanaka, R. Tanaka, S. Tanaka, R. Tanioka, B. B. Tannenwald, S. Tapia Araya, S. Tapprogge, S. Tarem, G. F. Tartarelli, P. Tas, M. Tasevsky, T. Tashiro, E. Tassi, A. Tavares Delgado, Y. Tayalati, A. C. Taylor, A. J. Taylor, G. N. Taylor, P. T. E. Taylor, W. Taylor, P. Teixeira-Dias, D. Temple, H. Ten Kate, P. K. Teng, J. J. Teoh, F. Tepel, S. Terada, K. Terashi, J. Terron, S. Terzo, M. Testa, R. J. Teuscher, S. J. Thais, T. Theveneaux-Pelzer, F. Thiele, J. P. Thomas, J. Thomas-Wilsker, P. D. Thompson, A. S. Thompson, L. A. Thomsen, E. Thomson, Y. Tian, M. J. Tibbetts, R. E. Ticse Torres, V. O. Tikhomirov, Yu. A. Tikhonov, S. Timoshenko, P. Tipton, S. Tisserant, K. Todome, S. Todorova-Nova, S. Todt, J. Tojo, S. Tokár, K. Tokushuku, E. Tolley, L. Tomlinson, M. Tomoto, L. Tompkins, K. Toms, B. Tong, P. Tornambe, E. Torrence, H. Torres, E. Torró Pastor, J. Toth, F. Touchard, D. R. Tovey, C. J. Treado, T. Trefzger, F. Tresoldi, A. Tricoli, I. M. Trigger, S. Trincaz-Duvoid, M. F. Tripiana, W. Trischuk, B. Trocmé, A. Trofymov, C. Troncon, M. Trottier-McDonald, M. Trovatelli, L. Truong, M. Trzebinski, A. Trzupek, K. W. Tsang, J. C.-L. Tseng, P. V. Tsiareshka, G. Tsipolitis, N. Tsirintanis, S. Tsiskaridze, V. Tsiskaridze, E. G. Tskhadadze, I. I. Tsukerman, V. Tsulaia, S. Tsuno, D. Tsybychev, Y. Tu, A. Tudorache, V. Tudorache, T. T. Tulbure, A. N. Tuna, S. Turchikhin, D. Turgeman, I. Turk Cakir, R. Turra, P. M. Tuts, G. Ucchielli, I. Ueda, M. Ughetto, F. Ukegawa, G. Unal, A. Undrus, G. Unel, F. C. Ungaro, Y. Unno, K. Uno, C. Unverdorben, J. Urban, P. Urquijo, P. Urrejola, G. Usai, J. Usui, L. Vacavant, V. Vacek, B. Vachon, K. O. H. Vadla, A. Vaidya, C. Valderanis, E. Valdes Santurio, M. Valente, S. Valentinetti, A. Valero, L. Valéry, S. Valkar, A. Vallier, J. A. Valls Ferrer, W. Van Den Wollenberg, H. van der Graaf, P. van Gemmeren, J. Van Nieuwkoop, I. van Vulpen, M. C. van Woerden, M. Vanadia, W. Vandelli, A. Vaniachine, P. Vankov, G. Vardanyan, R. Vari, E. W. Varnes, C. Varni, T. Varol, D. Varouchas, A. Vartapetian, K. E. Varvell, J. G. Vasquez, G. A. Vasquez, F. Vazeille, D. Vazquez Furelos, T. Vazquez Schroeder, J. Veatch, V. Veeraraghavan, L. M. Veloce, F. Veloso, S. Veneziano, A. Ventura, M. Venturi, N. Venturi, A. Venturini, V. Vercesi, M. Verducci, W. Verkerke, A. T. Vermeulen, J. C. Vermeulen, M. C. Vetterli, N. Viaux Maira, O. Viazlo, I. Vichou, T. Vickey, O. E. Vickey Boeriu, G. H. A. Viehhauser, S. Viel, L. Vigani, M. Villa, M. Villaplana Perez, E. Vilucchi, M. G. Vincter, V. B. Vinogradov, A. Vishwakarma, C. Vittori, I. Vivarelli, S. Vlachos, M. Vogel, P. Vokac, G. Volpi, H. von der Schmitt, E. von Toerne, V. Vorobel, K. Vorobev, M. Vos, R. Voss, J. H. Vossebeld, N. Vranjes, M. Vranjes Milosavljevic, V. Vrba, M. Vreeswijk, R. Vuillermet, I. Vukotic, P. Wagner, W. Wagner, J. Wagner-Kuhr, H. Wahlberg, S. Wahrmund, K. Wakamiya, J. Walder, R. Walker, W. Walkowiak, V. Wallangen, C. Wang, C. Wang, F. Wang, H. Wang, H. Wang, J. Wang, J. Wang, Q. Wang, R.-J. Wang, R. Wang, S. M. Wang, T. Wang, W. Wang, W. Wang, Z. Wang, C. Wanotayaroj, A. Warburton, C. P. Ward, D. R. Wardrope, A. Washbrook, P. M. Watkins, A. T. Watson, M. F. Watson, G. Watts, S. Watts, B. M. Waugh, A. F. Webb, S. Webb, M. S. Weber, S. M. Weber, S. W. Weber, S. A. Weber, J. S. Webster, A. R. Weidberg, B. Weinert, J. Weingarten, M. Weirich, C. Weiser, H. Weits, P. S. Wells, T. Wenaus, T. Wengler, S. Wenig, N. Wermes, M. D. Werner, P. Werner, M. Wessels, T. D. Weston, K. Whalen, N. L. Whallon, A. M. Wharton, A. S. White, A. White, M. J. White, R. White, D. Whiteson, B. W. Whitmore, F. J. Wickens, W. Wiedenmann, M. Wielers, C. Wiglesworth, L. A. M. Wiik-Fuchs, A. Wildauer, F. Wilk, H. G. Wilkens, H. H. Williams, S. Williams, C. Willis, S. Willocq, J. A. Wilson, I. Wingerter-Seez, E. Winkels, F. Winklmeier, O. J. Winston, B. T. Winter, M. Wittgen, M. Wobisch, A. Wolf, T. M. H. Wolf, R. Wolff, M. W. Wolter, H. Wolters, V. W. S. Wong, N. L. Woods, S. D. Worm, B. K. Wosiek, J. Wotschack, K. W. Wozniak, M. Wu, S. L. Wu, X. Wu, Y. Wu, T. R. Wyatt, B. M. Wynne, S. Xella, Z. Xi, L. Xia, D. Xu, L. Xu, T. Xu, W. Xu, B. Yabsley, S. Yacoob, D. Yamaguchi, Y. Yamaguchi, A. Yamamoto, S. Yamamoto, T. Yamanaka, F. Yamane, M. Yamatani, T. Yamazaki, Y. Yamazaki, Z. Yan, H. Yang, H. Yang, Y. Yang, Z. Yang, W-M. Yao, Y. C. Yap, Y. Yasu, E. Yatsenko, K. H. Yau Wong, J. Ye, S. Ye, I. Yeletskikh, E. Yigitbasi, E. Yildirim, K. Yorita, K. Yoshihara, C. Young, C. J. S. Young, J. Yu, J. Yu, S. P. Y. Yuen, I. Yusuff, B. Zabinski, G. Zacharis, R. Zaidan, A. M. Zaitsev, N. Zakharchuk, J. Zalieckas, A. Zaman, S. Zambito, D. Zanzi, C. Zeitnitz, G. Zemaityte, A. Zemla, J. C. Zeng, Q. Zeng, O. Zenin, T. Ženiš, D. Zerwas, D. Zhang, D. Zhang, F. Zhang, G. Zhang, H. Zhang, J. Zhang, L. Zhang, L. Zhang, M. Zhang, P. Zhang, R. Zhang, R. Zhang, X. Zhang, Y. Zhang, Z. Zhang, X. Zhao, Y. Zhao, Z. Zhao, A. Zhemchugov, B. Zhou, C. Zhou, L. Zhou, M. Zhou, M. Zhou, N. Zhou, Y. Zhou, C. G. Zhu, H. Zhu, J. Zhu, Y. Zhu, X. Zhuang, K. Zhukov, A. Zibell, D. Zieminska, N. I. Zimine, C. Zimmermann, S. Zimmermann, Z. Zinonos, M. Zinser, M. Ziolkowski, L. Živković, G. Zobernig, A. Zoccoli, R. Zou, M. zur Nedden, L. Zwalinski, ATLAS Collaboration

**Affiliations:** 1 Department of Physics, University of Adelaide, Adelaide, Australia; 2 Physics Department, SUNY Albany, Albany, NY USA; 3 Department of Physics, University of Alberta, Edmonton, AB Canada; 4 Department of Physics, Ankara University, Ankara, Turkey; 5 Istanbul Aydin University, Istanbul, Turkey; 6 Division of Physics, TOBB University of Economics and Technology, Ankara, Turkey; 7 LAPP, CNRS/IN2P3 and Université Savoie Mont Blanc, Annecy-le-Vieux, France; 8 High Energy Physics Division, Argonne National Laboratory, Argonne, IL USA; 9 Department of Physics, University of Arizona, Tucson, AZ USA; 10 Department of Physics, The University of Texas at Arlington, Arlington, TX USA; 11 Physics Department, National and Kapodistrian University of Athens, Athens, Greece; 12 Physics Department, National Technical University of Athens, Zografou, Greece; 13 Department of Physics, The University of Texas at Austin, Austin, TX USA; 14 Institute of Physics, Azerbaijan Academy of Sciences, Baku, Azerbaijan; 15 Institut de Física d’Altes Energies (IFAE), The Barcelona Institute of Science and Technology, Barcelona, Spain; 16 Institute of Physics, University of Belgrade, Belgrade, Serbia; 17 Department for Physics and Technology, University of Bergen, Bergen, Norway; 18 Physics Division, Lawrence Berkeley National Laboratory, University of California, Berkeley, CA USA; 19 Department of Physics, Humboldt University, Berlin, Germany; 20 Albert Einstein Center for Fundamental Physics and Laboratory for High Energy Physics, University of Bern, Bern, Switzerland; 21 School of Physics and Astronomy, University of Birmingham, Birmingham, UK; 22 Department of Physics, Bogazici University, Istanbul, Turkey; 23 Department of Physics Engineering, Gaziantep University, Gaziantep, Turkey; 24 Faculty of Engineering and Natural Sciences, Istanbul Bilgi University, Istanbul, Turkey; 25 Faculty of Engineering and Natural Sciences, Bahcesehir University, Istanbul, Turkey; 26 Centro de Investigaciones, Universidad Antonio Narino, Bogota, Colombia; 27 INFN Sezione di Bologna, Bologna, Italy; 28 Dipartimento di Fisica e Astronomia, Università di Bologna, Bologna, Italy; 29 Physikalisches Institut, University of Bonn, Bonn, Germany; 30 Department of Physics, Boston University, Boston, MA USA; 31 Department of Physics, Brandeis University, Waltham, MA USA; 32 Universidade Federal do Rio De Janeiro COPPE/EE/IF, Rio de Janeiro, Brazil; 33 Electrical Circuits Department, Federal University of Juiz de Fora (UFJF), Juiz de Fora, Brazil; 34 Federal University of Sao Joao del Rei (UFSJ), Sao Joao del Rei, Brazil; 35 Instituto de Fisica, Universidade de Sao Paulo, São Paulo, Brazil; 36 Physics Department, Brookhaven National Laboratory, Upton, NY USA; 37 Transilvania University of Brasov, Brasov, Romania; 38 Horia Hulubei National Institute of Physics and Nuclear Engineering, Bucharest, Romania; 39 Department of Physics, Alexandru Ioan Cuza University of Iasi, Iasi, Romania; 40 Physics Department, National Institute for Research and Development of Isotopic and Molecular Technologies, Cluj-Napoca, Romania; 41 University Politehnica Bucharest, Bucharest, Romania; 42 West University in Timisoara, Timisoara, Romania; 43 Departamento de Física, Universidad de Buenos Aires, Buenos Aires, Argentina; 44 Cavendish Laboratory, University of Cambridge, Cambridge, UK; 45 Department of Physics, Carleton University, Ottawa, ON Canada; 46 CERN, Geneva, Switzerland; 47 Enrico Fermi Institute, University of Chicago, Chicago, IL USA; 48 Departamento de Física, Pontificia Universidad Católica de Chile, Santiago, Chile; 49 Departamento de Física, Universidad Técnica Federico Santa María, Valparaiso, Chile; 50 Institute of High Energy Physics, Chinese Academy of Sciences, Beijing, China; 51 Department of Physics, Nanjing University, Nanjing, Jiangsu China; 52 Physics Department, Tsinghua University, Beijing, 100084 China; 53 University of Chinese Academy of Science (UCAS), Beijing, China; 54 Department of Modern Physics and State Key Laboratory of Particle Detection and Electronics, University of Science and Technology of China, Hefei, Anhui China; 55 School of Physics, Shandong University, Jinan, Shandong China; 56 School of Physics and Astronomy, Key Laboratory for Particle Physics, Astrophysics and Cosmology, Ministry of Education, Shanghai Key Laboratory for Particle Physics and Cosmology, Tsung-Dao Lee Institute, Shanghai Jiao Tong University, Shanghai, China; 57 Université Clermont Auvergne, CNRS/IN2P3, LPC, Clermont-Ferrand, France; 58 Nevis Laboratory, Columbia University, Irvington, NY USA; 59 Niels Bohr Institute, University of Copenhagen, Copenhagen, Denmark; 60 INFN Gruppo Collegato di Cosenza, Laboratori Nazionali di Frascati, Frascati, Italy; 61 Dipartimento di Fisica, Università della Calabria, Rende, Italy; 62 Faculty of Physics and Applied Computer Science, AGH University of Science and Technology, Kraków, Poland; 63 Marian Smoluchowski Institute of Physics, Jagiellonian University, Kraków, Poland; 64 Institute of Nuclear Physics, Polish Academy of Sciences, Kraków, Poland; 65 Physics Department, Southern Methodist University, Dallas, TX USA; 66 Physics Department, University of Texas at Dallas, Richardson, TX USA; 67 DESY, Hamburg and Zeuthen, Germany; 68 Lehrstuhl für Experimentelle Physik IV, Technische Universität Dortmund, Dortmund, Germany; 69 Institut für Kern- und Teilchenphysik, Technische Universität Dresden, Dresden, Germany; 70 Department of Physics, Duke University, Durham, NC USA; 71 SUPA-School of Physics and Astronomy, University of Edinburgh, Edinburgh, UK; 72 INFN e Laboratori Nazionali di Frascati, Frascati, Italy; 73 Fakultät für Mathematik und Physik, Albert-Ludwigs-Universität, Freiburg, Germany; 74 Departement de Physique Nucleaire et Corpusculaire, Université de Genève, Geneva, Switzerland; 75 INFN Sezione di Genova, Genoa, Italy; 76 Dipartimento di Fisica, Università di Genova, Genoa, Italy; 77 E. Andronikashvili Institute of Physics, Iv. Javakhishvili Tbilisi State University, Tbilisi, Georgia; 78 High Energy Physics Institute, Tbilisi State University, Tbilisi, Georgia; 79 II Physikalisches Institut, Justus-Liebig-Universität Giessen, Giessen, Germany; 80 SUPA-School of Physics and Astronomy, University of Glasgow, Glasgow, UK; 81 II Physikalisches Institut, Georg-August-Universität, Göttingen, Germany; 82 Laboratoire de Physique Subatomique et de Cosmologie, Université Grenoble-Alpes, CNRS/IN2P3, Grenoble, France; 83 Laboratory for Particle Physics and Cosmology, Harvard University, Cambridge, MA USA; 84 Kirchhoff-Institut für Physik, Ruprecht-Karls-Universität Heidelberg, Heidelberg, Germany; 85 Physikalisches Institut, Ruprecht-Karls-Universität Heidelberg, Heidelberg, Germany; 86 Faculty of Applied Information Science, Hiroshima Institute of Technology, Hiroshima, Japan; 87 Department of Physics, The Chinese University of Hong Kong, Shatin, NT Hong Kong; 88 Department of Physics, The University of Hong Kong, Hong Kong, China; 89 Department of Physics, Institute for Advanced Study, The Hong Kong University of Science and Technology, Clear Water Bay, Kowloon, Hong Kong, China; 90 Department of Physics, National Tsing Hua University, Taiwan, Taiwan; 91 Department of Physics, Indiana University, Bloomington, IN USA; 92 Institut für Astro- und Teilchenphysik, Leopold-Franzens-Universität, Innsbruck, Austria; 93 University of Iowa, Iowa City, IA USA; 94 Department of Physics and Astronomy, Iowa State University, Ames, IA USA; 95 Joint Institute for Nuclear Research, JINR Dubna, Dubna, Russia; 96 KEK, High Energy Accelerator Research Organization, Tsukuba, Japan; 97 Graduate School of Science, Kobe University, Kobe, Japan; 98 Faculty of Science, Kyoto University, Kyoto, Japan; 99 Kyoto University of Education, Kyoto, Japan; 100 Research Center for Advanced Particle Physics and Department of Physics, Kyushu University, Fukuoka, Japan; 101 Instituto de Física La Plata, Universidad Nacional de La Plata and CONICET, La Plata, Argentina; 102 Physics Department, Lancaster University, Lancaster, UK; 103 INFN Sezione di Lecce, Lecce, Italy; 104 Dipartimento di Matematica e Fisica, Università del Salento, Lecce, Italy; 105 Oliver Lodge Laboratory, University of Liverpool, Liverpool, UK; 106 Department of Experimental Particle Physics, Jožef Stefan Institute and Department of Physics, University of Ljubljana, Ljubljana, Slovenia; 107 School of Physics and Astronomy, Queen Mary University of London, London, UK; 108 Department of Physics, Royal Holloway University of London, Surrey, UK; 109 Department of Physics and Astronomy, University College London, London, UK; 110 Louisiana Tech University, Ruston, LA USA; 111 Laboratoire de Physique Nucléaire et de Hautes Energies, UPMC and Université Paris-Diderot and CNRS/IN2P3, Paris, France; 112 Fysiska institutionen, Lunds universitet, Lund, Sweden; 113 Departamento de Fisica Teorica C-15, Universidad Autonoma de Madrid, Madrid, Spain; 114 Institut für Physik, Universität Mainz, Mainz, Germany; 115 School of Physics and Astronomy, University of Manchester, Manchester, UK; 116 CPPM, Aix-Marseille Université and CNRS/IN2P3, Marseille, France; 117 Department of Physics, University of Massachusetts, Amherst, MA USA; 118 Department of Physics, McGill University, Montreal, QC Canada; 119 School of Physics, University of Melbourne, Melbourne, VIC Australia; 120 Department of Physics, The University of Michigan, Ann Arbor, MI USA; 121 Department of Physics and Astronomy, Michigan State University, East Lansing, MI USA; 122 INFN Sezione di Milano, Milan, Italy; 123 Dipartimento di Fisica, Università di Milano, Milan, Italy; 124 B.I. Stepanov Institute of Physics, National Academy of Sciences of Belarus, Minsk, Republic of Belarus; 125 Research Institute for Nuclear Problems of Byelorussian State University, Minsk, Republic of Belarus; 126 Group of Particle Physics, University of Montreal, Montreal, QC Canada; 127 P.N. Lebedev Physical Institute of the Russian Academy of Sciences, Moscow, Russia; 128 Institute for Theoretical and Experimental Physics (ITEP), Moscow, Russia; 129 National Research Nuclear University MEPhI, Moscow, Russia; 130 D.V. Skobeltsyn Institute of Nuclear Physics, M.V. Lomonosov Moscow State University, Moscow, Russia; 131 Fakultät für Physik, Ludwig-Maximilians-Universität München, Munich, Germany; 132 Max-Planck-Institut für Physik (Werner-Heisenberg-Institut), Munich, Germany; 133 Nagasaki Institute of Applied Science, Nagasaki, Japan; 134 Graduate School of Science and Kobayashi-Maskawa Institute, Nagoya University, Nagoya, Japan; 135 INFN Sezione di Napoli, Naples, Italy; 136 Dipartimento di Fisica, Università di Napoli, Naples, Italy; 137 Department of Physics and Astronomy, University of New Mexico, Albuquerque, NM USA; 138 Institute for Mathematics, Astrophysics and Particle Physics, Radboud University Nijmegen/Nikhef, Nijmegen, The Netherlands; 139 Nikhef National Institute for Subatomic Physics, University of Amsterdam, Amsterdam, The Netherlands; 140 Department of Physics, Northern Illinois University, DeKalb, IL USA; 141 Budker Institute of Nuclear Physics, SB RAS, Novosibirsk, Russia; 142 Department of Physics, New York University, New York, NY USA; 143 Ohio State University, Columbus, OH USA; 144 Faculty of Science, Okayama University, Okayama, Japan; 145 Homer L. Dodge Department of Physics and Astronomy, University of Oklahoma, Norman, OK USA; 146 Department of Physics, Oklahoma State University, Stillwater, OK USA; 147 Palacký University, RCPTM, Olomouc, Czech Republic; 148 Center for High Energy Physics, University of Oregon, Eugene, OR USA; 149 LAL, Univ. Paris-Sud, CNRS/IN2P3, Université Paris-Saclay, Orsay, France; 150 Graduate School of Science, Osaka University, Osaka, Japan; 151 Department of Physics, University of Oslo, Oslo, Norway; 152 Department of Physics, Oxford University, Oxford, UK; 153 INFN Sezione di Pavia, Pavia, Italy; 154 Dipartimento di Fisica, Università di Pavia, Pavia, Italy; 155 Department of Physics, University of Pennsylvania, Philadelphia, PA USA; 156 National Research Centre “Kurchatov Institute” B.P. Konstantinov Petersburg Nuclear Physics Institute, St. Petersburg, Russia; 157 INFN Sezione di Pisa, Pisa, Italy; 158 Dipartimento di Fisica E. Fermi, Università di Pisa, Pisa, Italy; 159 Department of Physics and Astronomy, University of Pittsburgh, Pittsburgh, PA USA; 160 Laboratório de Instrumentação e Física Experimental de Partículas-LIP, Lisbon, Portugal; 161 Faculdade de Ciências, Universidade de Lisboa, Lisbon, Portugal; 162 Department of Physics, University of Coimbra, Coimbra, Portugal; 163 Centro de Física Nuclear da Universidade de Lisboa, Lisbon, Portugal; 164 Departamento de Fisica, Universidade do Minho, Braga, Portugal; 165 Departamento de Fisica Teorica y del Cosmos, Universidad de Granada, Granada, Spain; 166 Dep Fisica and CEFITEC of Faculdade de Ciencias e Tecnologia, Universidade Nova de Lisboa, Caparica, Portugal; 167 Institute of Physics, Academy of Sciences of the Czech Republic, Prague, Czech Republic; 168 Czech Technical University in Prague, Prague, Czech Republic; 169 Faculty of Mathematics and Physics, Charles University, Prague, Czech Republic; 170 State Research Center Institute for High Energy Physics (Protvino), NRC KI, Protvino, Russia; 171 Particle Physics Department, Rutherford Appleton Laboratory, Didcot, UK; 172 INFN Sezione di Roma, Rome, Italy; 173 Dipartimento di Fisica, Sapienza Università di Roma, Rome, Italy; 174 INFN Sezione di Roma Tor Vergata, Rome, Italy; 175 Dipartimento di Fisica, Università di Roma Tor Vergata, Rome, Italy; 176 INFN Sezione di Roma Tre, Rome, Italy; 177 Dipartimento di Matematica e Fisica, Università Roma Tre, Rome, Italy; 178 Faculté des Sciences Ain Chock, Réseau Universitaire de Physique des Hautes Energies-Université Hassan II, Casablanca, Morocco; 179 Centre National de l’Energie des Sciences Techniques Nucleaires, Rabat, Morocco; 180 Faculté des Sciences Semlalia, Université Cadi Ayyad, LPHEA-Marrakech, Marrakech, Morocco; 181 Faculté des Sciences, Université Mohamed Premier and LPTPM, Oujda, Morocco; 182 Faculté des Sciences, Université Mohammed V, Rabat, Morocco; 183 DSM/IRFU (Institut de Recherches sur les Lois Fondamentales de l’Univers), CEA Saclay (Commissariat à l’Energie Atomique et aux Energies Alternatives), Gif-sur-Yvette, France; 184 Santa Cruz Institute for Particle Physics, University of California Santa Cruz, Santa Cruz, CA USA; 185 Department of Physics, University of Washington, Seattle, WA USA; 186 Department of Physics and Astronomy, University of Sheffield, Sheffield, UK; 187 Department of Physics, Shinshu University, Nagano, Japan; 188 Department Physik, Universität Siegen, Siegen, Germany; 189 Department of Physics, Simon Fraser University, Burnaby, BC Canada; 190 SLAC National Accelerator Laboratory, Stanford, CA USA; 191 Faculty of Mathematics, Physics and Informatics, Comenius University, Bratislava, Slovak Republic; 192 Department of Subnuclear Physics, Institute of Experimental Physics of the Slovak Academy of Sciences, Kosice, Slovak Republic; 193 Department of Physics, University of Cape Town, Cape Town, South Africa; 194 Department of Physics, University of Johannesburg, Johannesburg, South Africa; 195 School of Physics, University of the Witwatersrand, Johannesburg, South Africa; 196 Department of Physics, Stockholm University, Stockholm, Sweden; 197 The Oskar Klein Centre, Stockholm, Sweden; 198 Physics Department, Royal Institute of Technology, Stockholm, Sweden; 199 Departments of Physics and Astronomy and Chemistry, Stony Brook University, Stony Brook, NY USA; 200 Department of Physics and Astronomy, University of Sussex, Brighton, UK; 201 School of Physics, University of Sydney, Sydney, Australia; 202 Institute of Physics, Academia Sinica, Taipei, Taiwan; 203 Department of Physics, Technion: Israel Institute of Technology, Haifa, Israel; 204 Raymond and Beverly Sackler School of Physics and Astronomy, Tel Aviv University, Tel Aviv, Israel; 205 Department of Physics, Aristotle University of Thessaloniki, Thessaloníki, Greece; 206 International Center for Elementary Particle Physics and Department of Physics, The University of Tokyo, Tokyo, Japan; 207 Graduate School of Science and Technology, Tokyo Metropolitan University, Tokyo, Japan; 208 Department of Physics, Tokyo Institute of Technology, Tokyo, Japan; 209 Tomsk State University, Tomsk, Russia; 210 Department of Physics, University of Toronto, Toronto, ON Canada; 211 INFN-TIFPA, Trento, Italy; 212 University of Trento, Trento, Italy; 213 TRIUMF, Vancouver, BC Canada; 214 Department of Physics and Astronomy, York University, Toronto, ON Canada; 215 Faculty of Pure and Applied Sciences, and Center for Integrated Research in Fundamental Science and Engineering, University of Tsukuba, Tsukuba, Japan; 216 Department of Physics and Astronomy, Tufts University, Medford, MA USA; 217 Department of Physics and Astronomy, University of California Irvine, Irvine, CA USA; 218 INFN Gruppo Collegato di Udine, Sezione di Trieste, Udine, Italy; 219 ICTP, Trieste, Italy; 220 Dipartimento di Chimica, Fisica e Ambiente, Università di Udine, Udine, Italy; 221 Department of Physics and Astronomy, University of Uppsala, Uppsala, Sweden; 222 Department of Physics, University of Illinois, Urbana, IL USA; 223 Instituto de Fisica Corpuscular (IFIC), Centro Mixto Universidad de Valencia-CSIC, Valencia, Spain; 224 Department of Physics, University of British Columbia, Vancouver, BC Canada; 225 Department of Physics and Astronomy, University of Victoria, Victoria, BC Canada; 226 Department of Physics, University of Warwick, Coventry, UK; 227 Waseda University, Tokyo, Japan; 228 Department of Particle Physics, The Weizmann Institute of Science, Rehovot, Israel; 229 Department of Physics, University of Wisconsin, Madison, WI USA; 230 Fakultät für Physik und Astronomie, Julius-Maximilians-Universität, Würzburg, Germany; 231 Fakultät für Mathematik und Naturwissenschaften, Fachgruppe Physik, Bergische Universität Wuppertal, Wuppertal, Germany; 232 Department of Physics, Yale University, New Haven, CT USA; 233 Yerevan Physics Institute, Yerevan, Armenia; 234 Centre de Calcul de l’Institut National de Physique Nucléaire et de Physique des Particules (IN2P3), Villeurbanne, France; 235 Academia Sinica Grid Computing, Institute of Physics, Academia Sinica, Taipei, Taiwan; 236 CERN, 1211 Geneva 23, Switzerland

## Abstract

The inclusive and fiducial $t\bar{t}$ production cross-sections are measured in the lepton+jets channel using $20.2~\hbox {fb}^{-1}$ of proton–proton collision data at a centre-of-mass energy of 8 TeV recorded with the ATLAS detector at the LHC. Major systematic uncertainties due to the modelling of the jet energy scale and *b*-tagging efficiency are constrained by separating selected events into three disjoint regions. In order to reduce systematic uncertainties in the most important background, the $W \text {+\,jets}$ process is modelled using $Z$+ jets events in a data-driven approach. The inclusive $t\bar{t}$ cross-section is measured with a precision of 5.7% to be $\sigma _{\text {inc}}(t\bar{t}) = 248.3 \pm 0.7 \, ({\mathrm {stat.}}) \pm 13.4 \, ({\mathrm {syst.}}) \pm 4.7 \, ({\mathrm {lumi.}})~\text {pb}$, assuming a top-quark mass of 172.5 GeV. The result is in agreement with the Standard Model prediction. The cross-section is also measured in a phase space close to that of the selected data. The fiducial cross-section is $\sigma _{\text {fid}}(t\bar{t}) = 48.8 \pm 0.1 \, ({\mathrm {stat.}}) \pm 2.0 \, ({\mathrm {syst.}}) \pm 0.9 \, ({\mathrm {lumi.}})~\text {pb}$ with a precision of 4.5%.

## Introduction

The top quark is the most massive known elementary particle. Given that its Yukawa coupling to the Higgs boson is close to unity, it may play a special role in electroweak symmetry breaking [1, 2]. Studies of top-quark production and decay are major research goals at the LHC, providing both a precise probe of the Standard Model (SM) [3] and a window on physics beyond the Standard Model (BSM) [4]. The LHC supplies a large number of top-quark events to its major experiments, offering an excellent environment for such studies.

In proton–proton collisions, the dominant top-quark production process is pair production via the strong interaction. The measurement of the production cross-section provides a stringent test of QCD calculations with heavy quarks [5], allows a determination of the top-quark mass in a well-defined renormalisation scheme [6, 7], and can be sensitive to potential new physics such as top-quark partners degenerate in mass with the SM top quark [8].

The predicted inclusive $t\bar{t}$ cross-section at a centre-of-mass energy of $\sqrt{s} = 8~\hbox {TeV}$, assuming a top-quark mass $m_{\text {top}} = 172.5~\hbox {GeV}$, is

1 $$\begin{aligned} \sigma (pp \rightarrow t\bar{t}) \ = \ 253^{+13}_{-15}\ \ \ {{\mathrm {pb}}}. \end{aligned}$$

It is calculated at next-to-next-to-leading order (NNLO) in QCD including resummation of next-to-next-to-leading logarithmic (NNLL) soft-gluon terms with Top++ (v2.0) [5, 9–13]. The QCD scale uncertainties are determined as the maximum deviation in the predicted cross-section for the six probed variations following a prescription referred to as independent restricted scale variations. Here the renormalisation scale ($\mu _{\text {r}}$) and the factorisation scale ($\mu _{\text {f}}$) are varied independently to half the default scale and twice the default scale omitting the combinations ($0.5 \mu _{\text {r}}^{\text {def}}$, $2.0 \mu _{\text {f}}^{\text {def}}$) and ($2.0 \mu _{\text {r}}^{\text {def}}$, $0.5 \mu _{\text {f}}^{\text {def}}$). The uncertainties due to the parton distribution functions (PDFs) and $\alpha _{\text {S}}$ are calculated using the PDF4LHC prescription [14] where the uncertainties of the MSTW2008 68% CL NNLO [15, 16], CT10 NNLO [17, 18] and NNPDF 2.3  [19] PDF sets are added in quadrature to the $\alpha _{\text {S}}$ uncertainty. Comparable results are obtained using a different resummation technique as reported in Refs. [20, 21]. The predicted cross-section’s total scale, PDF and $\alpha _{\text {S}}$ uncertainty of about 6% sets the current goal for the experimental precision.

Measurements of the $t\bar{t}$ cross-section have been published for several centre-of-mass energies between 1.96 and 13 TeV in $p\bar{p}$ and *pp* collisions. At the Tevatron, the uncertainty in the $t\bar{t}$ cross-section measured by the D0 and CDF collaborations at a centre-of-mass energy of 1.96 TeV is 5.4% [22]. The most precise measurement for a centre-of-mass energy of 8 TeV, with a total uncertainty of 3.2%, was performed by the ATLAS Collaboration in the dilepton channel, where both top quarks decay via $t \rightarrow \ell \nu b$ [23]. The final-state charged lepton $\ell$ is either an electron or a muon.^1^ Further measurements at 7, 8 and 13 TeV in the same final state were published by the ATLAS and CMS collaborations [24–26]. Additionally, a measurement of the $t\bar{t}$ production cross-section in the forward region at 8 TeV was published by the LHCb collaboration [27].

The measurement reported in this paper is performed in the lepton+jets final state, where one *W* boson decays leptonically and the other *W* boson decays hadronically, i.e.

$$\begin{aligned} t\bar{t}\ \rightarrow W^+ W^- b\bar{b} \rightarrow \ell \nu q\bar{q}' b\bar{b}. \end{aligned}$$

Results are reported for both the full phase space and for a fiducial phase space close to the selected dataset.

Since experimental uncertainties may affect each decay mode differently, it is important to determine whether $t\bar{t}$ cross-sections measured in different decay modes are consistent with each other. Furthermore, new physics processes can contribute in different ways to the different decay modes.

The analysis is based on data collected at a *pp* centre-of-mass energy of $\sqrt{s} = 8~\hbox {TeV}$. The most precise cross-section previously measured in this channel at $\sqrt{s} = 8~\hbox {TeV}$ was published by the CMS Collaboration and reached an uncertainty of 6.8% [28]. This analysis supersedes the previous measurement from the ATLAS Collaboration, which achieved a total uncertainty of 9.4% using the same dataset [29]. This analysis improves on the previous result by splitting the overall sample of $t\bar{t}$ candidates into three signal regions and by constraining important sources of systematic uncertainty.

## ATLAS detector

The ATLAS detector [30] is a multi-purpose particle detector with forward-backward symmetry and a cylindrical geometry.^2^ The inner detector (ID) tracking system is surrounded by a thin superconducting solenoid magnet, electromagnetic and hadronic calorimeters, and a muon spectrometer (MS) in a magnetic field generated by three superconducting toroidal magnets of eight coils each. The inner detector, in combination with the 2 T magnetic field from the solenoid, provides precision momentum measurements for charged particles within the pseudorapidity range $\vert \eta \vert<$ 2.5. It consists of, from the interaction point to the outside, a silicon pixel detector and a silicon microstrip detector (together allowing a precise and efficient identification of secondary vertices), complemented with a straw-tube tracker contributing transition radiation measurements to electron identification. The calorimeter system covers the pseudorapidity range $\vert \eta \vert<$ 4.9. A high-granularity liquid-argon (LAr) sampling calorimeter with lead absorbers provides the measurement of electromagnetic showers within $\vert \eta \vert<$ 3.2. In the ID acceptance region, $\vert \eta \vert<$ 2.5, the innermost layer has a fine segmentation in $\eta$ to allow separation of electrons and photons from $\pi ^0$ decays and to improve the resolution of the shower position and direction measurements. Hadronic showers are measured by a steel/plastic-scintillator tile calorimeter in the central region, $\vert \eta \vert<$ 1.7, and by a LAr calorimeter in the endcap region, 1.5 $<\vert \eta \vert<$ 3.2. In the forward region, measurements of electromagnetic and hadronic showers are provided by a LAr calorimeter covering the pseudorapidity range 3.1 $<\vert \eta \vert<$ 4.9. The MS combines trigger and high-precision tracking detectors, and allows measurements of charged-particle trajectories within $\vert \eta \vert<$ 2.7. The combination of all ATLAS detector subsystems provides charged-particle tracking, along with identification for charged leptons and photons, in the pseudorapidity range $\vert \eta \vert<$ 2.5.

A three-level trigger system is used to select interesting events [31]. A hardware-based first-level trigger uses a subset of detector information to bring the event rate below 75 kHz. Two additional software-based trigger levels together reduce the event rate to about 400 Hz on average, depending on the data-taking conditions.

## Data and simulated events

This analysis is performed using *pp* collision data recorded at a centre-of-mass energy of $\sqrt{s}=8~\hbox {TeV}$, corresponding to the full 2012 dataset. The data-taking periods in which all the subdetectors were operational are considered, resulting in a data sample with an integrated luminosity of $\mathcal {L}_{\mathrm {int}} = \hbox {20.2 fb}^{-1}$.

Detector and trigger simulations were performed within the GEANT4 framework [32, 33]. The same offline reconstruction methods used on data are applied to the simulated events. Minimum-bias events generated with Pythia 8 [34] were used to simulate multiple *pp* interactions (pile-up). The distribution of the number of pile-up interactions in the simulation is reweighted according to the instantaneous luminosity spectrum in the data.

Signal $t\bar{t}$ events were simulated using the Powheg-Box event generator (r3026) [35, 36] with the CT10 PDF set [17]. The renormalisation and factorisation scales in the matrix element calculation were set to the value $\mu =\sqrt{m_{\text {top}} ^2 + p_{\text {T}} ^2(t)}$ where $p_{\text {T}} (t)$ is the top-quark transverse momentum, evaluated for the underlying Born configuration, i.e. before radiation. The $h_{\text {damp}}$ parameter, which controls the transverse momentum, $p_{\text {T}}$, of the first emission beyond the Born configuration, was set to $m_{\text {top}}$. The main effect of this is to regulate the high-$p_{\text {T}}$ gluon emission against which the $t\bar{t}$ system recoils. Parton shower (PS), hadronisation and the underlying event were simulated with Pythia  (v6.428) [37] and the Perugia2011C set of tuned parameters [38].

For systematic studies of the $t\bar{t}$ process, alternative event generators and variations of the tuned parameter values in Pythia are used. The Powheg-Box event generator, using the same configuration as the nominal sample, interfaced to Herwig (v6.5.20) [39] is used for hadronisation-modelling studies, while MC@NLO (v4.09) [40, 41] interfaced to Herwig is used to study the dependence on the matching method between the next-to-leading-order (NLO) matrix element (ME) generation and the PS evolution. In the case of events showered by Herwig, the Jimmy  (v4.31) [42] model with the ATLAS AUET2 [43] set of tuned parameters were used to simulate the underlying event. Variations of the amount of additional radiation are studied using events generated with the Powheg-Box + Pythia event generators after changing the scales in the ME and the scales in the parton shower simultaneously. In these samples, a variation of the factorisation and renormalisation scales by a factor of 2 was combined with the Perugia2012radLo parameters and a variation of both scale parameters by a factor of 0.5 was combined with the Perugia2012radHi parameters [38]. In the second case, the $h_{\text {damp}}$ parameter was also changed and set to twice the top-quark mass [44].

The associated production of an on-shell *W* boson and a top quark (*Wt*), and single top-quark production in the *s*- and *t*-channel, were simulated by the Powheg-Box (r2819, r2556) event generator [45, 46] with the CT10 PDF set interfaced to Pythia using the Perugia2011C set of tuned parameters. The *Wt* process has a predicted production cross-section of 22.3 pb [47], calculated to approximate NNLO accuracy with an uncertainty of 7.6% including scale and PDF uncertainties. The cross-sections for single top-quark production in the *s*- and *t*-channel are calculated with the Hathor v2.1 tool [48] to NLO precision, based on work documented in Ref. [49]. Uncertainties from variations of scales used in the ME and PDFs are estimated using the same methodology as for $t\bar{t}$ production. For *t*-channel production, this leads to a cross-section of 84.6 pb with a total uncertainty of 4.6%, while for *s*-channel production a cross-section of 5.2 pb with a total uncertainty of 4.2% is predicted.

All top-quark processes were simulated with a top-quark mass of 172.5 GeV and a width of 1.32 GeV modelled using a Breit–Wigner distribution. The top quark is assumed to decay via $t \rightarrow Wb$ 100% of the time.

Vector-boson production in association with jets (*W*/*Z*+jets) was simulated with Alpgen  (v2.14) [50], using the CTEQ6L1 set of PDFs [51]. The partonic events were showered with Pythia using the Perugia2011C set of tuned parameters. Simulated $W \text {+\,jets}$, $W+b\bar{b}, W+c\bar{c}, W+c$ and *Z*+jets, $Z+b\bar{b}, Z+c\bar{c}$ events with up to five additional partons were produced, and the overlap between the ME and the PS was removed with the “MLM” matching scheme [52]. The double-counting between the inclusive $W+n$ parton samples and dedicated samples with at least one heavy quark (*c*- or *b*-quark) in the ME was removed by vetoing events based on a $\Delta R$ matching. The cross-sections for inclusive *W*- and *Z*-boson production are calculated with NNLO precision using the FEWZ program [53, 54] and are estimated to be 12.1 and 1.13 nb, respectively. The uncertainty is 4%, including the contributions from the PDF and scale variations.

Samples of diboson (*VV*, $V=W$ or *Z*) events were produced using the Sherpa  (v1.4.1) event generator [55] with the CT10 PDF set, up to three additional partons in the ME, and a dedicated parton-shower tune developed by the Sherpa authors. The CKKW method [56] was used to remove overlap between partonic configurations generated by the ME or the PS. All three processes are normalised using the inclusive NLO cross-sections provided by MCFM  [57], which are 56.8 pb for *WW*, 7.36 pb for *ZZ*, and 21.5 pb for *WZ* production. The total uncertainty for each of the three processes, including scale variations and uncertainties in the PDF, is estimated to be 5%.

## Event reconstruction

In this analysis, $t\bar{t}$ candidate events are identified by means of isolated electrons and muons, jets, some of which are possibly *b*-tagged as likely to contain *b*-hadrons, and sizeable missing transverse momentum. The definitions of these reconstructed objects, called detector-level objects, together with the corresponding objects reconstructed using only MC event generator information, called particle-level objects, are discussed in this section. The particle-level objects are used to define a fiducial volume.

### Detector-level object reconstruction

*Electrons* Electron candidates are reconstructed by matching tracks in the ID to energy deposits (clusters) in the electromagnetic calorimeter [58]. Selected electrons are required to satisfy strict quality requirements in terms of shower shape, track properties and matching quality. Electron candidates are required to be within $|\eta | < 2.47$, and candidates in the calorimeter barrel–endcap overlap region, $1.37< |\eta | < 1.52$, are excluded. Electrons from heavy-flavour decays, hadronic jets misidentified as electrons, and photon conversions are the major backgrounds for high-$p_{\text {T}}$  electrons associated with a *W*-boson decay. The suppression of these backgrounds is possible via isolation criteria that require little calorimeter activity and a small sum of track $p_{\text {T}}$ in an $\eta$–$\phi$ cone around the electron. The electromagnetic (EM) calorimeter isolation variable is defined as the scalar sum of the transverse momenta of calorimeter energy deposits within the cone, corrected by subtracting the estimated contributions from the electron candidate and from the underlying event and pile-up contributions. The track isolation variable is defined as the scalar sum of all track transverse momenta within the cone, excluding the track belonging to the electron candidate [59]. Thresholds are imposed on the EM calorimeter isolation variable in a cone of size $\Delta R = 0.2$ around the electron and on the track isolation in a cone of size $\Delta R = 0.3$. The isolation requirements imposed on the electron candidates are tuned to achieve a uniform selection efficiency of 90% across electron transverse energy $E_{\text {T}}$ and pseudorapidity $\eta$. The electron pseudorapidity is taken from the associated track.

*Muons* Muon candidates are reconstructed by combining MS tracks with tracks in the ID, where tracks in the MS and ID are reconstructed independently [60]. The final candidates are required to be in the pseudorapidity region of $|\eta |<2.5$. A set of requirements on the number of hits in the ID must also be satisfied by muon candidates. An isolation requirement [61] is applied to reduce the contribution of muons from heavy-flavour decays. The isolation variable is defined as the scalar sum of the transverse momenta of all tracks originating from the primary vertex with $p_{\text {T}}$ above 1 GeV, excluding the one matched to the muon, within a cone of size $\Delta R_{\text {iso}}=10~\hbox {GeV}/p_{\text {T}} (\mu )$, where $p_{\text {T}} (\mu )$ is the transverse momentum of the muon. Muon candidates are accepted when the value of the isolation variable divided by the $p_{\text {T}} (\mu )$ is smaller than 0.05.

*Jets* Jets are reconstructed using the anti-$k_{t}$ algorithm [62] implemented in the FastJet package [63] with a radius parameter of 0.4, using topological clusters calibrated with the local cell weighting (LCW) method [64] as inputs to the jet finding algorithm. The energies of the reconstructed jets are calibrated using $p_{\text {T}}$- and $\eta$-dependent factors that are derived from MC simulation with a residual in situ calibration based on data [65]. In addition, a pile-up correction is applied to both the data and Monte Carlo (MC) events to further calibrate the jet before selection [66]. To reject jets likely to have originated from pile-up, a variable called the jet vertex fraction (${\mathrm {JVF}}$) is defined as the ratio of $\sum {p_{\mathrm {T},i\in \text {PV}}}$ of all tracks in the jet which originate from the primary vertex to the $\sum {p_{\mathrm {T},i}}$ of all tracks in the jet. Only tracks with $p_{\text {T}}$ > 1 GeV are considered in the ${\mathrm {JVF}}$ calculation. Jets with $|\eta |<2.4$ and $p_{\text {T}} < 50~\hbox {GeV}$ are required to have $|{\mathrm {JVF}}|> 0.5$.

*Identification of* *b*-*quark jets* One of the most important selection criteria for the analysis of events containing top quarks is the one that identifies jets likely to contain *b*-hadrons, called *b*-tagging. Identification of *b*-jets is based on the long lifetime of *b*-hadrons, which results in a significant flight path length and leads to reconstructable secondary vertices and tracks with large impact parameters relative to the primary vertex. In this analysis, a neural-network-based algorithm is used at a working point corresponding to a *b*-tagging efficiency in the simulated $t\bar{t}$ events of 70%, a *c*-jet rejection factor of 5 and light-flavour jet rejection factor of 140 [67].

*Missing transverse momentum* The missing transverse momentum is a measure of the momentum of the escaping neutrinos. It also includes energy losses due to detector inefficiencies, leading to the mismeasurement of the true transverse energy $E_{\text {T}}$ of the detected final-state objects. The missing transverse momentum vector, $\vec {E}_{\text {T}}^{\text {miss}}$, is calculated as the negative vector sum of the transverse momenta of reconstructed and calibrated physics objects, i.e. electrons, muons and jets as well as energy deposits in the calorimeter which are not associated with physics objects [68]. The magnitude of the missing transverse momentum vector is defined as $E_{\text {T}}^{\text {miss}} = | \vec {E}_{\text {T}}^{\text {miss}} |$.

A procedure to remove overlaps between physics objects is applied, where jets overlapping with identified electron candidates within a cone of size $\Delta R = 0.2$ are removed from the list of jets, as the jet and the electron are very likely to correspond to the same physics object. In order to ensure the selection of isolated charged leptons, further overlap removals are applied. If electrons are still present with distance $\Delta R < 0.4$ to a jet, they are removed from the event. Muons overlapping with a jet within $\Delta R < 0.4$ are discarded from the event.

### Particle-level object reconstruction

Particle-level objects are defined using stable particles with a mean lifetime greater than $0.3\cdot 10^{-10}~\hbox {s}$. Selected leptons are defined as electrons, muons or neutrinos originating from the *W*-boson decay, including those that originate from a subsequent $\tau$-lepton decay. Leptons from hadron decays either directly or via a hadronic $\tau$ decay are excluded. The selected charged lepton is combined with photons within $\Delta R < 0.1$, which implies that the final four-momentum vector is the vector sum of the associated photons and the original lepton four-vector. Finally the charged lepton is required to have $p_{\text {T}} > 25~\hbox {GeV}$ and $|\eta | < 2.5$.

Particle-level jets are reconstructed using the anti-$k_t$ algorithm with a radius parameter of $R=0.4$. All stable particles are used for jet clustering, except the selected leptons (electrons, muons or neutrinos) and the photons associated with the charged leptons. This implies that the energy of the particle level *b*-jet is close to that of the *b*-quark before hadronisation and fragmentation. Each jet is required to have $p_{\text {T}} > 25~\hbox {GeV}$ and $|\eta | < 2.5$.

Events are rejected if a selected lepton is at a distance $\Delta R < 0.4$ to a selected jet.

The fiducial volume is defined by selecting exactly one electron or muon and at least three particle-level jets. Setting the minimum number of particle-level jets to three minimises the extrapolation uncertainty going from the detector-level volume to the particle-level fiducial volume. In this case the fraction of events which are selected in the detector-level volume and not selected in the particle-level fiducial volume is of the order of 10%.

## Event selection and classification

This section describes the selection of $t\bar{t}$ candidate events. The datasets used in this analysis are obtained from single-electron or single-muon triggers. For the electron channel, a calorimeter energy cluster needs to be matched to a track, and the trigger electron candidate is required to have $E_{\text {T}} > 60~\hbox {GeV}$ or $E_{\text {T}} > 24~\hbox {GeV}$ with additional isolation requirements [31]. The single-muon trigger [69] requires either an isolated muon with $p_{\text {T}} >24~\hbox {GeV}$ or a muon with $p_{\text {T}} >36~\hbox {GeV}$.

Each event is required to have at least one vertex reconstructed from at least five tracks, where the $p_{\text {T}}$ of each track is above 400 MeV. The vertex with the largest sum of $p_{\text {T}}^{2}$ of the associated tracks is chosen as the primary vertex. Events containing any jets with $p_{\text {T}} > 20~\hbox {GeV}$ failing to satisfy quality criteria defined in Ref. [70] are rejected, in order to suppress background from beam–gas and beam-halo interactions, cosmic rays and calorimeter noise.

In order to select $t\bar{t}$ events in the lepton+jets channel, exactly one electron or muon with $p_{\text {T}} > 25~\hbox {GeV}$ is required. In addition to the requirements explained in Sect. 4, the $\Delta R$ value between the reconstructed lepton and the trigger-lepton has to be smaller than 0.15. Events containing an electron candidate and a muon candidate sharing an ID track are discarded.

Furthermore, events must have at least four jets with $p_{\text {T}} > 25~\hbox {GeV}$ and $|\eta |<2.5$. At least one of the jets has to be *b*-tagged. To enhance the fraction of events with a leptonically decaying *W* boson, events are required to have $E_{\text {T}}^{\text {miss}} > 25~\hbox {GeV}$ and the transverse mass $m_{\text {T}}(W)$ of the lepton–$E_{\text {T}}^{\text {miss}}$ pair is required to be

$$\begin{aligned} m_{\text {T}}(W)= & {} \sqrt{2 p_{\text {T}} (\ell ) \cdot E_{\text {T}}^{\text {miss}} \left[ 1-\cos \left( \Delta \phi \left( \vec {\ell }, \vec {E}_{\text {T}}^{\text {miss}} \right) \right) \right] }\\> & {} 30~\hbox {GeV}, \end{aligned}$$

where $p_{\text {T}} (\ell )$ is the transverse momentum of the charged lepton and $\Delta \phi$ is the angle in the transverse plane between the charged lepton and the $\vec {E}_{\text {T}}^{\text {miss}}$ vector.

The measurement of the $t\bar{t}$ cross-section is performed by splitting the selected sample into three disjoint signal regions. These have different sensitivities to the various backgrounds, to the production of additional radiation, and to detector effects.

- SR1: $\ge 4$ jets, 1 *b*-tag
  In this region, events with at least four jets of which exactly one is *b*-tagged are selected. This region has the highest background fraction of all three signal regions, with $W \text {+\,jets}$ being the dominant background. This signal region has the highest number of selected events.
- SR2: 4 jets, 2 *b*-tags
  In this region, events with exactly four jets of which exactly two are *b*-tagged are selected. The background is expected to be small in this region and this allows an unambiguous matching of the reconstructed objects to the top-quark decay products. In particular, the two untagged jets are likely to originate from the hadronically decaying *W* boson. The reconstructed *W*-boson mass is sensitive to the jet energy scale and to additional radiation.
- SR3: $\ge 4$ jets, $\ge 2$ *b*-tags (excluding events from SR2)
  In the third region, events are required to have at least four jets with at least two *b*-tagged jets. Events with exactly four jets and two *b*-tags are assigned to SR2. This region includes $t\bar{t}$ events with extra gluon radiation, including $t\bar{t}$ + heavy-flavour production, and is sensitive to the efficiency of misidentifying *c*-jets, originating mainly from the $W\rightarrow cs$ decay, as *b*-jets. The expected background is the smallest among the signal regions.

For the determination of the $t\bar{t}$ cross-section a discriminating variable in each signal region is defined, as explained in Sect. 7. The number of $t\bar{t}$ events is extracted using a simultaneous fit of three discriminating-variable distributions, one from each signal region, to data. In order to reduce systematic uncertainties due to the jet energy scale and *b*-tagging efficiency, their effects on the signal and background distributions are parameterised with nuisance parameters, which are included in the fit.

## Background modelling and estimation

The dominant background to $t\bar{t}$ production in the lepton+jets final state is $W \text {+\,jets}$ production. This analysis uses a sample defined from data to model the shapes of the discriminating-variable distributions for this background, while the normalisation in each signal region is determined in the final fit. The multijet background process, which is difficult to model in the simulation, is also modelled using data and normalised using control regions. All remaining backgrounds are determined using simulated events and theoretical predictions.

The method used to model the $W \text {+\,jets}$ background shape from data is based on the similarity of the production and decay of the *Z* boson to those of the *W* boson.

First, an almost background-free $Z$+ jets sample is selected in the following way:

- Events are required to contain exactly two oppositely charged leptons of the same flavour, i.e. $e^{+} e^{-}$ or $\mu ^{+} \mu ^{-}$, and
- the dilepton invariant mass $m(\ell \ell )$ has to be consistent with the *Z*-boson mass ($80< m(\ell \ell ) < 102~\hbox {GeV}$).

These events are then ‘converted’ into $W \text {+\,jets}$ events. This is achieved by boosting the leptons of the *Z*-boson decay into the *Z* boson’s rest frame, scaling their momenta to that of a lepton decay from a *W* boson by the ratio of the boson masses and boosting the leptons back into the laboratory system. The scaled lepton momenta are given by

$$\begin{aligned} \vec {p'^{*}}_{\ell _i}=\frac{m_W}{m_Z} \vec {p^{*}}_{\ell _i}, \end{aligned}$$

where $\vec {p^{*}}_{\ell _i}$ is the momentum vector of lepton *i* in the *Z*-boson’s rest frame, $m_W$ and $m_Z$ are the masses of the *W*- and *Z*-bosons respectively, and $\vec {p'^{*}}_{\ell _i}$ is the scaled momentum vector of lepton *i* in the *Z*-boson’s rest frame.

After this conversion, one of the leptons is randomly chosen to be removed, and the $\vec {E}_{\text {T}}^{\text {miss}}$ vector is recalculated. Finally, the event selection requirements discussed in Sect. 5 are applied, except for the *b*-tagging requirement. In the following, this sample is referred to as the $Z$ to $W$ sample.

**Fig. 1:**
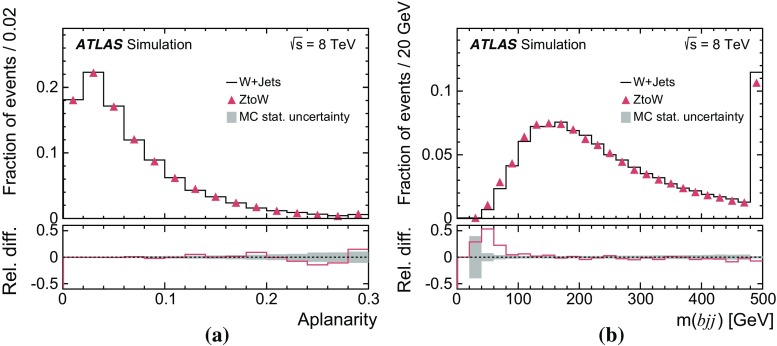
Probability densities of **a** the aplanarity and **b** the mass distribution of the hadronically decaying top-quark candidates for simulated $W \text {+\,jets}$ events with at least four jets and at least one *b*-tag and $Z$ to $W$ events derived from a simulated $Z$+ jets sample with at least four jets and no *b*-tagging requirement. The lower histogram shows the relative difference between the numbers of $Z$ to $W$ and $W \text {+\,jets}$ events in each bin with respect to the number of $W \text {+\,jets}$ events. The grey error band represents the Monte Carlo statistical uncertainty of the $W \text {+\,jets}$ sample. Events beyond the *x*-axis range are included in the last bin

Detailed studies in simulation and in validation regions are performed. As an example, two important variables, discriminating between $W \text {+\,jets}$ and $t\bar{t}$ events, are compared between simulated $W \text {+\,jets}$ events with at least four jets and at least one *b*-tag and $Z$ to $W$ events derived from a simulated $Z$+ jets sample with at least four jets and no *b*-tagging requirement. Distributions of these variables are shown in Fig. 1: the aplanarity event-shape variable and the mass of the hadronically decaying top-quark candidate. Details about the top-quark reconstruction are given in Sect. 7. The aplanarity is defined as

2 $$\begin{aligned} A= & {} \frac{3}{2}\lambda _3, \end{aligned}$$

where $\lambda _3$ is the smallest eigenvalue of the sphericity tensor, defined by

$$\begin{aligned} S^{\alpha \beta }= & {} \frac{\sum _i p_i^{\alpha } p_i^{\beta } }{\sum _i\mid p_i \mid ^2 }. \end{aligned}$$

Here, $\alpha$ and $\beta$ correspond to the *x*, *y* and *z* momentum components of final-state object *i* in the event, i.e. the jets, the charged lepton and the reconstructed neutrino (see Sect. 7).

Residual differences between the shapes of the $W \text {+\,jets}$ and $Z$ to $W$ templates are accounted for as a systematic uncertainty in the analysis. Since the method only provides shape information, the expected number of events for the $W \text {+\,jets}$ process in the signal regions is obtained from the acceptance of simulated samples using Alpgen + Pythia and normalised using the inclusive NNLO $W \text {+\,jets}$ cross-section as described in Sect. 3. These numbers are used to define the nominal background yield prior to the fit and used as initial values for the fit in the final statistical analysis.

Multijet events may be selected if a jet is misidentified as an isolated lepton or if the event has a non-prompt lepton that appears to be isolated (these two sources of background are referred to as fake leptons). The normalisation of the multijet background is obtained from a fit to the observed $E_{\text {T}}^{\text {miss}}$ distribution in the electron channel or to the $m_{\text {T}}(W)$  distribution in the muon channel in the signal regions. In order to construct a sample of multijet background events, different methods are adopted for the electron and muon channels.

The ‘jet-lepton’ method [71] is used to model the background due to fake electrons using a dijet sample simulated with the Pythia 8 event generator [34]. A jet that resembles the electron has to have $E_{\text {T}} > 25~\hbox {GeV}$ and be located in the same $\eta$ region as the signal electrons. The jet energy must have an electromagnetic fraction of between 0.8 and 0.95. The event is accepted if exactly one such jet is found, and if the event passes all other selection requirements as described above, except the one on $E_{\text {T}}^{\text {miss}}$. The yield of the multijet background in the electron-triggered data sample is then estimated using a binned maximum-likelihood fit to the $E_{\text {T}}^{\text {miss}}$ distribution using the template determined from the selected events in the dijet simulated sample. In order to improve the modelling of the $\eta (\ell )$ distribution of the ‘jet-lepton’ model in SR1, the fit is done separately in the barrel region ($|\eta | \le 1.37$) and in the endcap region ($|\eta | > 1.52$). The fits for SR2 and SR3 are performed inclusively in $|\eta |$ due to the lower number of selected events.

The ‘anti-muon’ method [71] uses a dedicated selection on data to enrich a sample in events that contain fake muons in order to build a multijet model for muon-triggered events. Some of the muon identification requirements differ from those for signal muon candidates. The calorimeter isolation requirement is inverted, while keeping the total energy loss of the muon in the calorimeters below $6~\hbox {GeV}$, and the requirement on the impact parameter is omitted. The additional application of all other event selection requirements mentioned in Sect. 5 results in a sample that is highly enriched in fake muons from multijet events, but contains only a small fraction of prompt muons from *Z*- and *W*-boson decays. The yield of the multijet background in the muon-triggered data sample is estimated from a maximum-likelihood fit to the $m_{\text {T}}(W)$ distribution using the template determined from the selected multijet events in the data sample. A different fit observable ($m_{\text {T}}(W)$) in the muon channel is used, since it provides a better modelling of the multijet background than the $E_{\text {T}}^{\text {miss}}$ observable used in the electron channel.

**Fig. 2:**
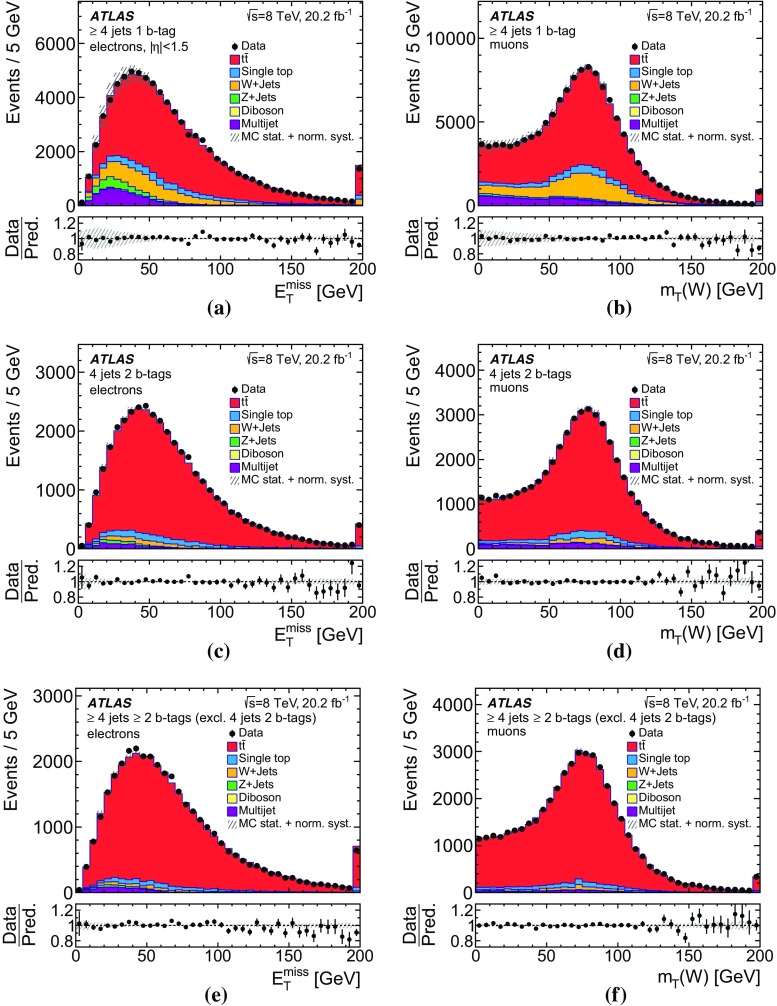
Observed and simulated (left) $E_{\text {T}}^{\text {miss}}$ distributions in the electron channel and (right) $m_{\text {T}}(W)$ distributions in the muon channel, normalised to the result of the binned maximum-likelihood fit, **a** for the barrel region in SR1, **b** in SR1, **c**, **d** in SR2, and **e**, **f** in SR3. The hatched error bands represent the uncertainty due to the sample size and the normalisation of the backgrounds. The ratio of observed to predicted (Pred.) number of events in each bin is shown in the lower histogram. Events beyond the *x*-axis range are included in the last bin

In both methods to obtain the multijet background normalisation, the multijet template is fitted together with templates derived from MC simulation for the $t\bar{t}$ and $W \text {+\,jets}$ processes. The $t\bar{t}$ and $W \text {+\,jets}$ rate uncertainties, obtained from theoretical cross-section uncertainties, are accounted for in the fitting process in the form of constrained normalisation factors. The rates for $Z$+ jets, single-top-quark processes, and *VV* processes are fixed to the predictions as described in Sect. 3. For the fits in SR2 and SR3, the $W \text {+\,jets}$ process is fixed as well, since the predicted yield is very small in these signal regions. The resulting fitted rate of $t\bar{t}$ events is in agreement within the statistical uncertainty with the result of the final estimation of the $t\bar{t}$ cross-section and therefore does not bias the result. Distributions of the fitted observable, normalised to the fit results, are shown in Fig. 2.

The ‘matrix’ method [71] is used as an alternative method to estimate systematic uncertainties in the multijet background estimate. It provides template distributions and estimates of the number of multijet events in SR1. Differences between the fitting method and the ‘matrix’ method are taken into account as systematic uncertainties yielding a normalisation uncertainty of 67%. Due to the small number of events for the ‘matrix’ method in SR2 and SR3 an uncertainty of 50% is assigned, based on comparisons of the rates obtained using alternative methods described in previous analyses [71].

As a result of the above procedure, the fraction of the total background estimated to originate from multijet events for $E_{\text {T}}^{\text {miss}} > 25~\hbox {GeV}$ and $m_{\text {T}}(W) > 30~\hbox {GeV}$ is $(5.4\pm 3.0)\%$ in SR1, $(2.6\pm 1.3)\%$ in SR2 and $(1.5\pm 0.8)\%$ in SR3. All other processes, namely $t\bar{t}$ and single top-quark production, $Z$+ jets and *VV* production, are modelled using simulation samples as described in Sect. 3.

Table 1 summarises the event yields in the three signal regions for the $t\bar{t}$ signal process and each of the background processes. The yields, apart from the multijet background, are calculated using the acceptance from MC samples normalised using their respective theoretical cross-sections as discussed in Sect. 3. The quoted uncertainties correspond to the statistical uncertainties in the Monte Carlo samples, except in the case of the multijet background where they correspond to the uncertainties in the background estimate.

**Table 1:** Event yields for the three signal regions. The multijet background and its uncertainty are estimated from the $E_{\text {T}}^{\text {miss}}$ or $m_{\text {T}}(W)$ fit to data. All the other expectations are derived using theoretical cross-sections, and the corresponding Monte Carlo sample statistical uncertainty

Process	SR1	SR2	SR3
$t\bar{t}$	$133\,310\pm 370$	$63\,060\pm 250$	$59\,310\pm 240$
Single top	$11\,020\pm 110$	$3728 \pm 61$	$2593 \pm 51$
$W \text {+\,jets}$	$29 \,870\pm 170$	$2382 \pm 49$	$1592 \pm 40$
$Z$+ jets	$3569 \pm 60$	$406 \pm 20$	$270 \pm 16$
Diboson	$1339 \pm 37$	$135 \pm 12$	$112 \pm 11$
Multijet	$10\,300\pm 6900$	$1940\pm 970$	$1050\pm 530$
Total expected	$189\,400\pm 6900$	$71\,700\pm 1000$	$64\,920\pm 580$
Observed	192 686	72 978	70 120

## Discriminating observables

In order to further separate the signal events from background events in SR1 and SR3, the output distribution of an artificial neural network (NN) [72, 73] is used. A large number of potential NN input variables are studied for their discriminating power between $W \text {+\,jets}$ and $t\bar{t}$ and the compatibility of their distributions between simulated $W \text {+\,jets}$ events with at least one *b*-tag and $Z$ to $W$ events with no *b*-tagging requirement. The observables investigated are based on invariant masses of jets and leptons, event shape observables and properties of the reconstructed top quarks.

In SR1 and SR3, the semileptonically decaying top quark is reconstructed. First, the leptonically decaying *W* boson’s four-momentum is reconstructed from the identified charged lepton’s four-momentum and the $E_{\text {T}}^{\text {miss}}$ vector, the latter representing the transverse momentum of the neutrino. The unmeasured *z*-component of the neutrino momentum $p_z(\nu )$ is inferred by imposing a *W*-boson mass constraint on the lepton–neutrino system, leading to a two-fold ambiguity. In the case of two real solutions, the one with the lower $|p_z(\nu )|$ is chosen. In the case of complex solutions, which can occur due to the finite $E_{\text {T}}^{\text {miss}}$ resolution, a fit is performed that rescales the neutrino $p_x$ and $p_y$ such that the imaginary radical vanishes, at the same time keeping the transverse components of the neutrino’s momentum as close as possible to the *x*- and *y*-components of $E_{\text {T}}^{\text {miss}}$. To reconstruct the semileptonically decaying top quark, the four jets with the highest $p_{\text {T}}$ are selected and the one with the smallest $\Delta R$ to the charged lepton is chosen to be the *b*-jet. The semileptonically decaying top quark is then reconstructed by adding the four-momentum of the *W* boson and the chosen *b*-jet. The hadronically decaying top quark is reconstructed by adding the four-momenta of the remaining three highest-$p_{\text {T}}$ jets.

**Table 2:** List of the seven input variables of the NN, ordered by their discriminating power

Variable	Definition
$m_{12}$	The smallest invariant mass between jet pairs
$\cos (\theta ^*)_{bjj}$	Cosine of the angle between the hadronic top-quark momentum and the beam direction in the $t\bar{t}$ rest frame
$m(\ell \nu b)$	Mass of the reconstructed semileptonically decaying top quark
*A*	Aplanarity, as defined in Eq. (2)
*m*(*bjj*)	Mass of the reconstructed hadronically decaying top quark
$m_{\ell 1}$	The smallest invariant mass between the charged lepton and a jet
$m_{23}$	The second smallest invariant mass between jet pairs

Seven observables are finally chosen as input variables to the NN (see Table 2). The choice was made by studying the correlations between the potential input variables and choosing the ones with small correlations that still provide a good separation between the signal and the background events. The NN infrastructure consists of one input node for each input variable plus one bias node, eight nodes in the hidden layer, and one output node, which gives a continuous output $o_{\mathrm {NN}}$ in the interval [0, 1]. For the training of the NN, an equal number of simulated $t\bar{t}$ events and $Z$ to $W$ events are used. The training is performed in an inclusive phase space with $\ge 4$ jets and $\ge 1$ *b*-tag to cover the whole phase space and achieve an optimal separation power in both signal regions.

**Fig. 3:**
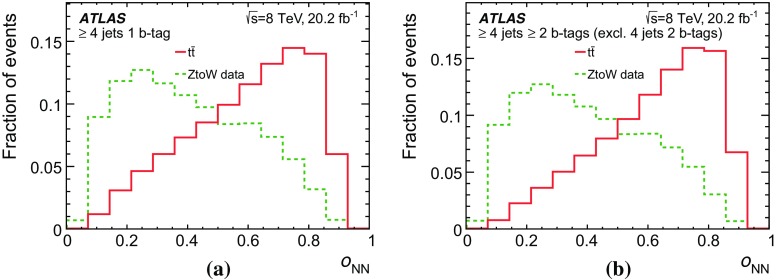
Probability densities of the neural-network discriminant $o_{\mathrm {NN}}$ for the simulated $t\bar{t}$ signal process and the $W \text {+\,jets}$ background process derived from data using converted $Z$+ jets events **a** for SR1 and **b** for SR3

The discriminating power of the NN between $Z$ to $W$ and $t\bar{t}$ events can be seen in Fig. 3 for SR1 and SR3.

**Fig. 4:**
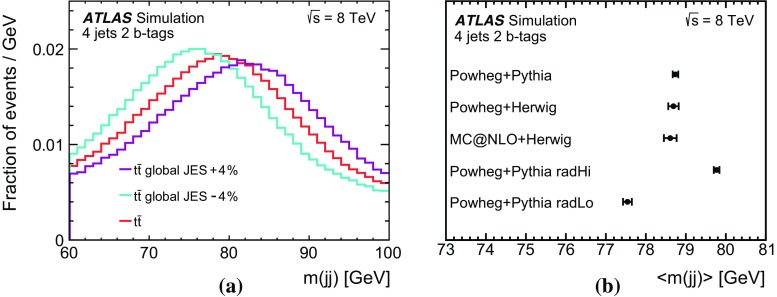
**a** Probability densities of the *m*(*jj*) distribution from the $t\bar{t}$ signal process for three different values of the JES, where events beyond the *x*-axis range are not shown and the range is restricted to show the peak. **b** Mean value of the fit to the *m*(*jj*) distribution using a Gaussian distribution for different signal generator set-ups. The uncertainties shown are statistical only

In SR2, a different distribution is used as discriminant in the final fit. Inspired by measurements of the top-quark mass, where the invariant mass of the two untagged jets *m*(*jj*) is frequently utilised to reduce the impact of the jet energy scale (JES) uncertainty [74–77], this approach is also followed here. The dependency of *m*(*jj*) on the JES is shown in Fig. 4a using simulated $t\bar{t}$ events with modified global JES correction factors. Here the energy of the jets is scaled by $\pm 4\%$. Additionally, the mean of the *m*(*jj*) distribution is sensitive to the amount of additional radiation. A comparison of the mean value of a Gaussian distribution fitted to the *m*(*jj*) distribution in the range of $60~\hbox {GeV}< m(jj) < 100~\hbox {GeV}$ for different generator set-ups is presented in Fig. 4b. It can be seen that the mean value is compatible for different generator set-ups, but varies for different settings of the parameters controlling the initial- and final-state radiation. For these reasons, the *m*(*jj*) observable is used as the discriminant in SR2.

Finally, the ratio of single to double *b*-tagged events, i.e. the ratio of events in SR1 to the sum of events in SR2 and SR3, is sensitive to the *b*-tagging efficiency. The effect of the *b*-tagging efficiency is parameterised with a nuisance parameter in the final fit. Since only two *b*-jets are present in $t\bar{t}$ events, any additional *b*-tagged jets originate either from heavy-flavour production in the parton shower or from mistagged *c*-hadrons. The inclusion of events with more than two *b*-tags in SR3 gives a small sensitivity to heavy-flavour production in the parton shower.

## Sources and estimation of systematic uncertainties

Several sources of systematic uncertainty affect the $t\bar{t}$ cross-section measurement. In addition to the luminosity determination, they are related to the modelling of the physics objects, the modelling of $t\bar{t}$ production and the understanding of the background processes. All of them affect the yields and kinematic distributions (shape of the distributions) in the three signal regions.

### Physics objects modelling

Systematic uncertainties associated with reconstructed jets, electrons and muons, due to residual differences between data and MC simulations after calibration, and uncertainties in corrective scale factors are propagated through the entire analysis.

Uncertainties due to the lepton trigger, reconstruction and selection efficiencies in simulation are estimated from measurements of the efficiency in data using $Z\rightarrow \ell \ell$ decays. The same processes are used to estimate uncertainties in the lepton momentum scale and resolution, and correction factors and their associated uncertainties are derived to match the simulated distributions to the observed distributions [58–60].

**Fig. 5:**
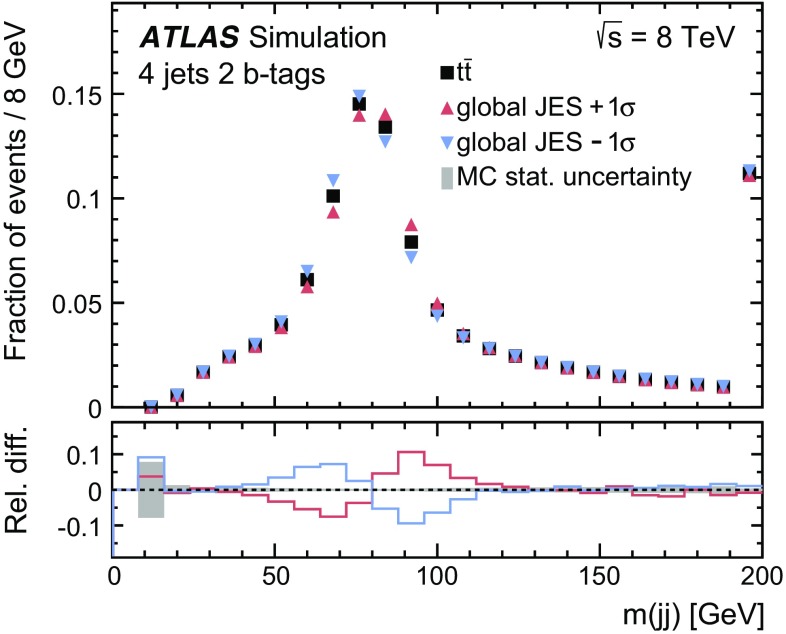
Probability density of the *m*(*jj*) distribution from simulated $t\bar{t}$ events in SR2 for the nominal JES and the $\pm 1 \sigma$ variation. The lower histogram shows the relative difference between the numbers of $t\bar{t}$ events for the $\pm 1 \sigma$ JES and the nominal JES in each bin with respect to the nominal JES. The grey error band represents the statistical uncertainty of the sample. Events beyond the *x*-axis range are included in the last bin

The JES uncertainties are derived using information from test-beam data, collision data and simulation. The uncertainty is parameterised in terms of jet $p_{\text {T}}$ and $\eta$ [65]. The JES uncertainty is broken down into various components originating from the calibration method, the calorimeter response, the detector simulation, and the set of parameters used in the MC event generator. Furthermore, contributions from the modelling of pile-up effects, differences between jets induced by *b*-quarks and those from gluons or light-quarks are included. A large uncertainty in the JES originates from the a-priori unknown relative fractions of quark-induced and gluon-induced jets in a generic sample, which is normally assumed to be $(50\pm 50)\%$. In this analysis, the actual fraction of gluon-induced jets is estimated in simulated events, which leads to a reduction in the uncertainty of these components by half. The fraction of gluon-induced jets is obtained, considering all selected jets apart from *b*-jets and it is between 15% to 30% depending on the $p_{\text {T}}$ and $\eta$ of the jet. The uncertainty in this fraction is estimated by comparing different $t\bar{t}$ samples, namely Powheg-Box + Pythia, Powheg-Box + Herwig, and MC@NLO + Herwig as well as samples with varied scale settings in the Powheg-Box + Pythia set-up. To estimate the systematic uncertainty of the JES, a parameterisation with 25 uncorrelated components is used, as described in Ref. [65]. For the purpose of the extraction of the $t\bar{t}$ cross-section, a single correction factor for the JES is included in the fit as a nuisance parameter (see Sect. 9). In this procedure, the dependence of the acceptance and the shape of the *m*(*jj*) template distribution on the JES is parameterised using the global JES uncertainty correction factor corresponding to the total JES uncertainty. Figure 5 shows the effect of a $\pm 1 \sigma$ change in the global JES correction factor on the *m*(*jj*) distribution. When estimating the systematic uncertainty in the $t\bar{t}$ cross-section due to the JES in the statistical procedure, all 25 components are considered and evaluated as described in Sect. 9.

Smaller uncertainties originate from modelling of the jet energy resolution [78, 79] and missing transverse momentum [68] to account for contributions from pile-up, soft jets, and calorimeter cells not matched to any jets. Uncertainties from the scale and resolution corrections for leptons and jets are propagated into the calculation of the missing transverse momentum as well. The effect of uncertainties associated with the ${\mathrm {JVF}}$ is also considered for each jet.

Since the analysis makes use of *b*-tagging, the uncertainties in the *b*-tagging efficiencies and the *c*-jet and light-jet mistag probabilities are taken into account [80, 81]. Correction factors applied to simulated events compensate for differences between data and simulation in the tagging efficiency for *b*-jets, *c*-jets and light-flavour jets. The correction for *b*-jets is derived from $t\bar{t}$ events in the dilepton channel and dijet events, and are found to be consistent with unity with uncertainties at the level of a few percent over most of the jet $p_{\text {T}}$ range [81]. Similar to the JES, the uncertainty in the correction factor of the *b*-tagging efficiency is included as a nuisance parameter in the fit for the extraction of the $t\bar{t}$ cross-section. The parameterisation of the correction factor is obtained from the total uncertainty in the *b*-tagging efficiency.

### Signal Monte Carlo modelling and parton distribution functions

Systematic effects from MC modelling are estimated by comparing different event generators and varying parameters for the event generation of the signal process.

The uncertainty from renormalisation and factorisation scale variations, and amount of additional radiation in the parton shower is estimated using the Powheg-Box event generator interfaced to Pythia by varying these scales and using alternative sets of tuned parameters for the parton shower as described in Sect. 3. Systematic effects due to the matching of the NLO matrix-element calculation and the parton shower for $t\bar{t}$ is estimated by comparing MC@NLO with Powheg-Box, both interfaced to the Herwig parton shower. An uncertainty related to the modelling of parton-shower, hadronisation effects and underlying-event, is estimated by comparing samples produced with Powheg-Box + Herwig and Powheg-Box + Pythia. More details about these samples are given in Sect. 3.

Systematic uncertainties related to the PDF set are taken into account for the signal process. The uncertainty is calculated following the PDF4LHC recommendation [82] using the PDF4LHC15_NLO PDF set. In addition, the acceptance difference between PDF4LHC15_NLO and CT10 is considered, since the latter PDF set is not covered by the uncertainty obtained with PDF4LHC15_NLO and it is used in the simulation of $t\bar{t}$ events. This uncertainty is used in the final results, since it is larger than the uncertainty obtained with PDF4LHC15_NLO.

Finally, the statistical uncertainty of the MC samples as well as the $Z$ to $W$ data sample is included.

### Background normalisation for non-fitted backgrounds

Uncertainties in the normalisation of the non-fitted backgrounds, i.e. single-top-quark, *VV*, and $Z$+ jets events, are estimated using the uncertainties in the theoretical cross-section predictions. In the case of $Z$+ jets, an uncertainty of 24% per additional jet is added to the uncertainty of the inclusive cross-section in quadrature leading to an total uncertainty of 48% for events with four jets. The uncertainty in the multijet background is obtained in SR1 from the comparison between the fitting method and the ‘matrix’ method as detailed in Sect. 6. For the other two regions, an uncertainty of 50% is used.

### Background modelling

Uncertainties in the shape of the $W \text {+\,jets}$ and multijet backgrounds are taken into account for the discriminating observables used in the analysis. For the $W \text {+\,jets}$ background, shape uncertainties are extracted from the differences between *Z*-boson and *W*-boson production. Although their production modes are very similar, differences exist in the details of the production and decay. There are differences in heavy-flavour production and in the helicity structures of the decay vertices. Shape variations are built from a comparison of the NN discriminant and the *m*(*jj*) distribution between simulated $W \text {+\,jets}$ events, described in Sect. 3, and $Z$ to $W$ events derived from a simulated $Z$+ jets sample. The uncertainty in the multijet background kinematics is estimated from the differences between the predictions from the ‘jet-lepton’ or ‘anti-muon’ method and the ‘matrix’ method in SR1.

### Luminosity

The absolute luminosity scale is derived from beam-separation scans performed in November 2012. The uncertainty in the integrated luminosity is 1.9% [83].

### Beam energy

The beam energy of the LHC was determined at 4 TeV from the LHC magnetic model together with measurements of the revolution frequency difference of proton and lead-ion beams, with an uncertainty of 0.1% [84]. The impact of the uncertainty of the beam energy on the measured cross-section is negligible.

## Extraction of the $t\bar{t}$ cross-section

The measured inclusive cross-section is given by

3 $$\begin{aligned} \sigma _{\mathrm {inc}} = \frac{\hat{\nu }}{\epsilon \cdot \mathcal {L}_{\mathrm {int}}} = \frac{\hat{\beta } \cdot \nu }{\epsilon \cdot \mathcal {L}_{\mathrm {int}}} \quad \text {with} \ \epsilon = \frac{N_{\mathrm {sel}}}{N_{\mathrm {total}}}, \end{aligned}$$

where $\hat{\nu }$ is the observed number of signal events. The quantity $\epsilon$ is the total event-selection efficiency, $N_{\mathrm {total}}$ is the number of events obtained from a simulated signal sample before applying any requirement and $N_{\mathrm {sel}}$ is the number of events obtained from the same simulated signal sample after applying all selection requirements. In practice, $\hat{\nu }$ is given by $\hat{\beta } \cdot \nu$, where $\hat{\beta }$ is an estimated scale factor obtained from a binned maximum-likelihood fit and $\nu = \epsilon \cdot \sigma _{\mathrm {theo}} \cdot \mathcal {L}_{\mathrm {int}}$ is the expected number of events for the signal process. The reference cross-section $\sigma _{\mathrm {theo}}$ is defined by the central value of the theoretical prediction given in Eq. (1). By combining Eq. (3) with the expected number of events, one obtains:

$$\begin{aligned} \sigma _{\mathrm {inc}} = \hat{\beta } \cdot \sigma _{\mathrm {theo}}. \end{aligned}$$

The fiducial cross-section is given by

$$\begin{aligned} \sigma _{\mathrm {fid}} = A_{\mathrm {fid}}\cdot \sigma _{\mathrm {inc}} \quad \text {with} \ A_{\mathrm {fid}} = \frac{N_{\mathrm {fid}}}{N_{\mathrm {total}}}, \end{aligned}$$

with $N_{\mathrm {fid}}$ being the number of events obtained from a simulated signal sample after applying the particle-level selection. Here $A_{\mathrm {fid}}$ is defined for an inclusive $t\bar{t}$ sample, including all decay modes of the *W* bosons. Using Eq. (3), the fiducial cross-section can be written as:

4 $$\begin{aligned} \sigma _{\mathrm {fid}} = \frac{\hat{\nu }}{\epsilon ^{\prime } \cdot \mathcal {L}_{\mathrm {int}}} \quad \text {with}\ \epsilon ^{\prime } = \frac{N_{\mathrm {sel}}}{N_{\mathrm {fid}}}. \end{aligned}$$

From Eq. (4) it is apparent that signal modelling uncertainties that affect $N_{\mathrm {sel}}$ and $N_{\mathrm {fid}}$ in a similar way give a reduced uncertainty in $\sigma _{\mathrm {fid}}$ compared to that in $\sigma _{\mathrm {inc}}$.

The binned maximum-likelihood fit is performed simultaneously in the three signal regions defined in Sect. 5. For SR1 and SR3 the distribution used in the fit is the NN discriminant, while the invariant-mass distribution *m*(*jj*) of the two untagged jets is used in SR2. Electron- and muon-triggered events are combined in the templates used in this fit.

Scale factors $\beta ^{t\bar{t}}$ and $\beta ^{W_j}$ for the signal and $W \text {+\,jets}$ background, respectively, and two nuisance parameters $\delta _i$, namely the *b*-tagging efficiency correction factor $\delta _{b-{\mathrm {tag}}}$ and the JES correction factor $\delta _{\mathrm {JES}}$, are fitted in all three signal regions simultaneously. The $\delta _i$ are defined such that 0 corresponds to the nominal value and $\pm 1$ to a deviation of $\pm 1 \sigma$ of the corresponding systematic uncertainty.

In order to account for differences in the flavour composition of the $W \text {+\,jets}$ background, two uncorrelated scale factors are used: one in SR1 ($\beta ^{W_1}$) and one in the two other signal regions ($\beta ^{W_{2,3}}$). The event yields of the other backgrounds are not allowed to vary in the fit, but instead are fixed to their predictions. The likelihood function is given by the product of the Poisson likelihoods in the individual bins *M* of the histograms. A Gaussian prior is incorporated into the likelihood function to constrain $\delta _{b-{\mathrm {tag}}}$ within the associated uncertainty:

$$\begin{aligned}&L(\beta ^{t\bar{t}}, \beta ^{W_1}, \beta ^{W_{2,3}} ,\delta _{b\text {-tag}},\delta _{\mathrm {JES}})\\&\quad =\prod _{k=1}^M \frac{\mathrm {e}^{-\mu _k}\cdot \mu _k^{n_k}}{n_k!} \;\cdot \; G(\delta _{b\text {-tag}}; 0, 1) \end{aligned}$$

with

$$\begin{aligned} \mu _k= & {} \beta ^s \cdot \nu _{s} \cdot \alpha ^s_k + \sum _{j=1}^2 \beta ^{W_j} \cdot \nu _{W_j} \cdot \alpha ^{W_j}_k + \sum _{b=1}^4 \nu _{b} \cdot \alpha ^{b}_k, \\ \beta ^s= & {} \beta ^{t\bar{t}} \left\{ 1 + \sum _{i=1}^2 |\delta _i| \cdot \left( H(\delta _i)\cdot \epsilon _{i+} + H(-\delta _i)\cdot \epsilon _{i-} \right) \right\} , \\ \alpha ^s_k= & {} \alpha ^{t\bar{t}}_k \sum _{i=1}^2 |\delta _i| \cdot \left\{ (\alpha _{ki}^+ - \alpha _{k})\cdot H(\delta _i) + (\alpha _{ki}^- - \alpha _{k})\cdot H(-\delta _i) \right\} . \end{aligned}$$

The expected number of events $\mu _k$ in bin *k* is the sum of the expected number of events for the signal and the background processes. These are given by the product of the predicted number of events $\nu _p$ of each process and the fraction of events $\alpha ^{p}_k$ in bin *k* of the normalised distribution. Here *p* denotes the signal *s* and background processes $W_j$ and *b*, where *b* represents the background processes which are not varied in the fit. The number of events observed in bin *k* is denoted by $n_k$. For the $t\bar{t}$ signal, the scale factor $\beta ^s$ contains the acceptance uncertainties for positive $\epsilon _{i+}$ and negative $\epsilon _{i-}$ variations of the two profiled systematic uncertainties, multiplied by their nuisance parameter $\delta _i$. The symbol *H* denotes the Heaviside function. The signal template shape for each profiled systematic variation is calculated by interpolating in each bin *k* between the standard template $\alpha _{k}$ and the systematically altered histograms $\alpha _{ki}^{\pm }$ using the nuisance parameter $\delta _i$ as a weight. Linearity and closure tests are done to validate the statistical procedure.

The fit found the minimum of the negative log-likelihood function for the parameter values shown in Table 3. The estimators for the nuisance parameters, which parameterise their optimal shift relative to the default value 0 in terms of their uncertainty, are found to be $\hat{\delta } = 0.62\pm$ 0.09 for the *b*-tagging efficiency correction factor and $0.68\pm 0.07$ for JES correction factor. This deviation of the *b*-tagging efficiency correction factor from the nominal value of the simulated sample corresponds to a shift of the acceptance in SR1 of 1% and 2.6% in SR2 and SR3. The deviation for the JES correction factor corresponds to a shift of the acceptance of 2.9% in SR1, of 1.4% in SR2, and of 4.4% in SR3. The deviation of the JES correction factor also potentially accounts for differences in the modelling of additional radiation between the different MC event generator set-ups. Finally, the fitted scale factor of the $W \text {+\,jets}$ process in SR2 and SR3 yields a value significantly higher than the one predicted by MC simulation, consistent with previous measurements indicating an underestimate of heavy-flavour production in the simulation [85].

**Table 3:** Result of the maximum-likelihood fit to data. Estimators of the parameters of the likelihood function, the scale factor $\hat{\beta }$ for the $t\bar{t}$ and the two $W \text {+\,jets}$ channels and the derived contributions of the various processes to the three signal regions are listed. Only the statistical uncertainties obtained from the maximum-likelihood fit are shown for $t\bar{t}$ and $W \text {+\,jets}$, while the normalisation uncertainties are quoted for the other processes

Process	$\hat{\beta }$	SR1	SR2	SR3
$t\bar{t}$	$0.982 \pm 0.005$	$133\,390 \pm 630$	$64\,360\pm 300$	$62\,380\pm 280$
$W \text {+\,jets}$ 1 *b*-tag	$1.08 \pm 0.02$	$32\,150\pm 480$	–	–
$W \text {+\,jets}$ $\ge$ 2 *b*-tags	$1.41 \pm 0.08$	–	$3370\pm 190$	$2250 \pm 130$
Single top	–	$11\,020\pm 660$	$3730\pm 220$	$2590\pm 160$
$Z$+ jets	–	$3600\pm 1700$	$410\pm 200$	$270 \pm 130$
Diboson	–	$1300\pm 640$	$135 \pm 65$	$112 \pm 54$
Multijet	–	$10\, 300 \pm 6900$	$1940 \pm 970$	$1050 \pm 530$
Total sum	–	$191\,700 \pm 7200$	$73\, 900 \pm 1100$	$68\,660 \pm 650$
Total observed	–	192686	72978	70120

**Fig. 6:**
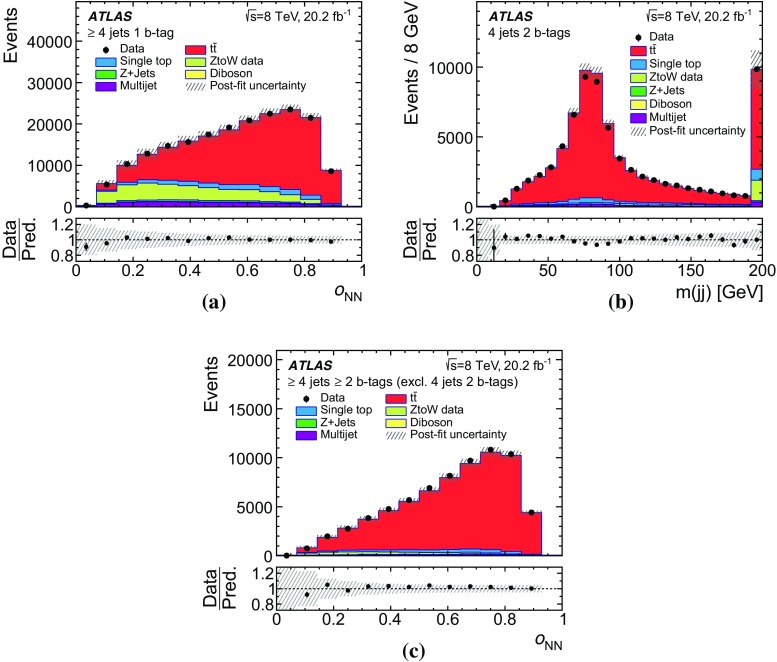
Neural network discriminant $o_{\mathrm {NN}}$ or the *m*(*jj*) distribution normalised to the result of the maximum-likelihood fit for **a** SR1, **b** SR2, and **c** SR3. The hatched error bands represent the post-fit uncertainty. The ratio of observed to predicted (Pred.) number of events in each bin is shown in the lower histogram. Events beyond the *x*-axis range are included in the last bin

**Fig. 7:**
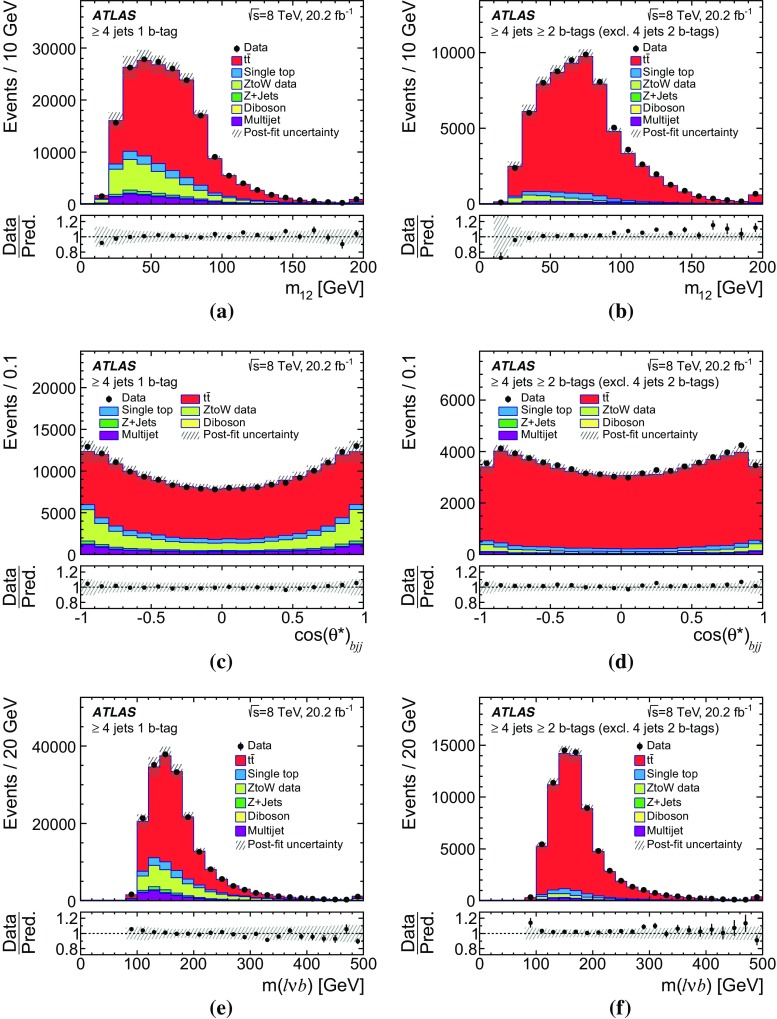
Distributions of the three most discriminating NN input variables for (left) SR1 and (right) SR3. The signal and backgrounds are normalised to the result of the maximum-likelihood fit: **a**, **b** smallest invariant mass between jet pairs, **c**, **d** cosine of the angle between the hadronic-top-quark momentum and the beam direction in the $t\bar{t}$ rest frame, and **e**, **f** mass of the reconstructed semileptonically decaying top quark. The hatched error bands represent the post-fit uncertainty. The ratio of observed to predicted (Pred.) number of events in each bin is shown in the lower histogram. Events beyond the *x*-axis range are included in the last bin

The signal and background templates scaled and morphed to the fitted values of the fit parameters are compared to the observed distributions of the NN discriminant in SR1 and SR3 and the *m*(*jj*) distribution in SR2, shown in Fig. 6. Comparisons of the data and the fit results are shown for the three most discriminating input variables of the NN in Fig. 7 for SR1 and for SR3.

The systematic uncertainties in the cross-section measurements are estimated using pseudo-experiments. In each of these experiments, the detector effects, background contributions and model uncertainties are varied within their systematic uncertainties. They impact the yields of the processes and shapes of the template distributions used to create the pseudo-datasets in the three signal regions. Correlations between rate and shape uncertainties for a given component are taken into account. The entire set of pseudo-experiments can thereby be interpreted as a replication of the sample space of all systematic variations. By measuring the $t\bar{t}$ cross-section, an estimator of the probability density of all possible outcomes of the measurement is obtained. The RMS of this estimator distribution is itself an estimator of the observed uncertainties. Using the measured $t\bar{t}$ cross-section and the estimated nuisance parameters, the uncertainty of the actual measurement is estimated.

The total uncertainty in both the inclusive and the fiducial $t\bar{t}$ cross-section is presented in Table 4 and is estimated to be $5.7\%$ for the inclusive measurement and $4.5\%$ for the fiducial measurement. The breakdown of the contributions from individual, or categories of, systematic uncertainties are also listed. In this case, only the considered source or group of sources is varied in the generation of the pseudo-experiments. The largest uncertainty in the inclusive measurement is due to the uncertainty in the PDF sets and the MC modelling of the signal process. The effects of uncertainties in the JES and the *b*-tagging efficiency have been significantly reduced by including them as nuisance parameters together with the chosen signal regions and discriminant distributions. Furthermore, the uncertainty due to additional radiation is reduced by a factor of three thanks to the inclusion of the *m*(*jj*) distribution in the analysis. For the fiducial cross-section measurement, the uncertainties in the MC modelling and PDF sets are reduced. The uncertainty in the $t\bar{t}$ cross-section due to the PDF sets is largest for events which are produced in the forward direction, i.e. one initial gluon has a high Bjorken-*x* value. The PDFs for high-*x* gluons have large uncertainties in all current PDF sets. Selecting events in the fiducial volume reduces the fraction of such events significantly and therefore the uncertainty is reduced significantly as well. In the case of the MC modelling, the uncertainty due to additional radiation is reduced more than the parton-shower and NLO-matching uncertainties, since varying the amount of radiation leads to similar changes in the selection efficiencies of the fiducial and reconstructed volumes and therefore to smaller uncertainties in the $t\bar{t}$ cross-section.

**Table 4:** Breakdown of relative uncertainties in the measured inclusive and fiducial $t\bar{t}$ cross-sections. The total uncertainties contain all considered uncertainties

Source	$\frac{\Delta \sigma _{\text {inc}}}{\sigma _{\text {inc}}}$ [%]	$\frac{\Delta \sigma _{\text {fid}}}{\sigma _{\text {fid}}}$ [%]
Statistical uncertainty	0.3	0.3
*Physics object modelling*		
Jet energy scale	1.1	1.1
Jet energy resolution	0.1	0.1
Jet reconstruction efficiency	$< 0.1$	$< 0.1$
$E_{\text {T}}^{\text {miss}}$ scale	0.1	0.1
$E_{\text {T}}^{\text {miss}}$ resolution	$< 0.1$	$< 0.1$
Muon momentum scale	$< 0.1$	$< 0.1$
Muon momentum resolution	$< 0.1$	$< 0.1$
Electron energy scale	0.1	0.1
Electron energy resolution	$< 0.1$	$< 0.1$
Lepton identification	1.4	1.4
Lepton reconstruction	0.3	0.3
Lepton trigger	1.3	1.3
*b*-tagging efficiency	0.3	0.3
*c*-tagging efficiency	0.5	0.5
Mistag rate	0.3	0.3
*Signal Monte Carlo modelling and parton distribution functions*		
NLO matching	1.1	0.9
Scale variations	2.2	1.0
Parton shower	1.3	0.9
PDF	3.0	0.1
*Background normalisation for non-fitted backgrounds*		
Single top	0.3	0.3
$Z$+ jets	0.2	0.2
Diboson	0.1	0.1
*Background modelling*		
$Z$ to $W$ modelling	1.1	1.1
Multijet	0.6	0.6
Luminosity	1.9	1.9
Total (syst.)	5.7	4.5
Total (syst. + stat.)	5.7	4.5

## Results

After performing a binned maximum-likelihood fit to the NN discriminant distributions and the *m*(*jj*) distribution, and estimating the total uncertainty, the inclusive $t\bar{t}$ cross-section is measured to be:

$$\begin{aligned} \sigma _{\text {inc}}(t\bar{t}) = 248.3 \pm 0.7 \, ({\mathrm {stat.}}) \pm 13.4 \, ({\mathrm {syst.}}) \pm 4.7 \, ({\mathrm {lumi.}}) \ {\mathrm {pb}} \end{aligned}$$

assuming a top-quark mass of $m_{\text {top}} = 172.5~\hbox {GeV}$.

The fiducial cross-section measured in the fiducial volume defined in Sect. 4.2 with acceptance $A_{\text {fid}}=19.6\%$ is:

$$\begin{aligned} \sigma _{\text {fid}}(t\bar{t}) = 48.8 \pm 0.1 \, ({\mathrm {stat.}}) \pm 2.0 \, ({\mathrm {syst.}}) \pm 0.9 \, ({\mathrm {lumi.}})~\text {pb}. \end{aligned}$$

The dependence of the inclusive $t\bar{t}$ cross-section measurement on the assumed value of $m_{\text {top}}$ is mainly due to acceptance effects and can be expressed by the function:

$$\begin{aligned} \sigma _{t\bar{t}}(m_{\text {top}}) = \sigma _{t\bar{t}}(172.5~\hbox {GeV}) + p_1 \cdot \Delta m_{\text {top}} + p_2 \cdot \Delta m_{\text {top}} ^{2}, \end{aligned}$$

with $\Delta m_{\text {top}} = m_{\text {top}}- 172.5~\hbox {GeV}$. The parameters $p_1=-2.07 \pm 0.07~\hbox {pb}/\hbox {GeV}$ and $p_2=0.07 \pm 0.02~\hbox {pb}/\hbox {GeV}^2$ are determined using dedicated signal samples with different $m_{\text {top}}$ values, where signal template distributions are obtained from the alternative samples and the fit to data is repeated.

A combination of the cross-section in this channel with the more precise result in the dilepton channel [86] was tested. The central values of the two results are consistent within 0.2%, but due to the higher precision of the dilepton result, the combination yielded only a marginal improvement.

## Conclusions

A measurement of both the inclusive and fiducial $t\bar{t}$ cross-sections in *pp* collisions at $\sqrt{s} = 8~\hbox {TeV}$ in the lepton+jets channel is presented using data collected in 2012 with the ATLAS detector at the LHC, corresponding to an integrated luminosity of $20.2~\hbox {fb}^{-1}$.

In order to reduce major uncertainties coming from the jet energy scale and the *b*-tagging efficiency, the analysis splits the selected data sample into three disjoint signal regions with different numbers of *b*-tagged jets and different jet multiplicities. Using an artificial neural network, the separation between the signal and background processes is improved compared to using single observables. Additionally, the analysis makes use of a data-driven approach to model the dominant $W \text {+\,jets}$ background. It is modelled from collision data by converting $Z$+ jets candidate events into a $W \text {+\,jets}$ sample.

The $t\bar{t}$ cross-section is determined using a binned maximum-likelihood fit to the three signal regions, constraining correction factors for the jet energy scale and the *b*-tagging efficiency. The inclusive $t\bar{t}$ cross-section is measured with a precision of 5.7% to be:

$$\begin{aligned} \sigma _{\text {inc}}(t\bar{t}) = 248.3 \pm 0.7 \, ({\mathrm {stat.}}) \pm 13.4 \, ({\mathrm {syst.}}) \pm 4.7 \, ({\mathrm {lumi.}}) \ {\mathrm {pb}} \end{aligned}$$

assuming a top-quark mass of $m_{\text {top}} = 172.5~\hbox {GeV}$.

The fiducial cross-section is measured with a precision of 4.5% to be:

$$\begin{aligned} \sigma _{\text {fid}}(t\bar{t}) = 48.8 \pm 0.1 \, ({\mathrm {stat.}}) \pm 2.0 \, ({\mathrm {syst.}}) \pm 0.9 \, ({\mathrm {lumi.}})~\text {pb}. \end{aligned}$$

This result is a significant improvement on the previous ATLAS measurement at $\sqrt{s} = 8~\hbox {TeV}$ in the lepton+jets channel and is in agreement with measurements of the inclusive $t\bar{t}$ cross-section in other decay modes and with the theoretical prediction.

## Footnotes

1 Events involving $W\rightarrow \tau \nu$ decays with a subsequent decay of the $\tau$ lepton to either $e\nu _e\nu _\tau$ or $\mu \nu _\mu \nu _\tau$ are included in the signal.

2 ATLAS uses a right-handed coordinate system with its origin at the nominal interaction point (IP) in the centre of the detector and the *z*-axis along the beam pipe. The *x*-axis points from the IP to the centre of the LHC ring, and the *y*-axis points upwards. Cylindrical coordinates $(r,\phi )$ are used in the transverse plane, $\phi$ being the azimuthal angle around the *z*-axis. The pseudorapidity is defined in terms of the polar angle $\theta$ as $\eta = -\ln \tan (\theta /2)$, and the distance $\Delta R$ in the $\eta$–$\phi$ space is defined as $\Delta R \equiv \sqrt{(\Delta \eta )^{2} + (\Delta \phi )^{2}}$.
